# Comparative Acetylation and Ubiquitination Profiling in Cervical Cancer-Cell Models Supports a Proposed Lysine-Fate Code

**DOI:** 10.3390/ijms27219749

**Published:** 2026-10-30

**Authors:** Ramiro Alonso Bastida, Marisol Ayala Reyes, György Marko-Varga, Jeovanis Gil, Sergio Encarnación-Guevara

**Affiliations:** 1 Centro de Ciencias Genómicas, Universidad Nacional Autónoma de México (UNAM), Cuernavaca 62210, Mexico; ramiro.alonb@gmail.com (R.A.B.); marayala@ccg.unam.mx (M.A.R.); 2 Section for Clinical Chemistry, Department of Translational Medicine, Lund University, 22242 Lund, Sweden; gyorgy.marko-varga@bme.lth.se (G.M.-V.); jeovanis.gil_valdes@med.lu.se (J.G.); 3 Department of Biotechnology, College of Life Science and Biotechnology, Yonsei University, Seoul 03722, Republic of Korea; 4 1st Department of Surgery, Tokyo Medical University, Tokyo 160-8582, Japan

**Keywords:** lysine acetylation, ubiquitination, post-translational modifications, cervical cancer, stoichiometry, DIA mass spectrometry, HPV, proteostasis, PTM crosstalk, proteomics

## Abstract

Post-translational modifications are rarely studied as an integrated system. Most studies have focused on acetylation or ubiquitination in isolation, despite both frequently targeting the same lysine residues on the same proteins. This study investigated whether the interplay between these modifications, rather than their individual effects, constitutes a meaningful regulatory layer in cervical cancer cells. Quantitative stoichiometric proteomics was applied to HPV-positive and HPV-negative cell models, with a non-cancerous keratinocyte cell line serving as the control. The results demonstrate that acetylation and ubiquitination form coordinated, context-dependent patterns rather than acting independently. In HPV-positive cells acetylation predominated and was enriched in transcriptional regulators. Conversely, the HPV-negative C33A line exhibited a substantially larger ubiquitinome, indicative of increased proteostatic surveillance. Site-specific stoichiometry analysis revealed that the relative occupancy of each modification at shared lysine residues determines regulatory outcomes, rather than their mere presence. This competition is proposed to constitute a “lysine-fate code” that integrates transcriptional regulation, metabolic adaptation, and immune signaling into a unified proteostatic logic. Beyond their implications for cervical cancer biology, these findings suggest that the acetylation–ubiquitination balance may represent a key regulatory principle. Targeting enzymes that modulate this balance could provide novel therapeutic strategies that surpass single-pathway inhibition.

## 1. Introduction

Cervical cancer is the fourth most prevalent cancer among women worldwide. In 2018, there were over 570,000 new cases and 311,000 deaths. The disease has a higher impact on women in low- and middle-income countries. Limited access to screening and treatment leads to higher incidence and mortality [1,2]. Persistent infection with high-risk human papillomavirus (HPV), especially HPV16 and HPV18, is the leading cause of cervical cancer [3,4]. These viruses drive malignant transformation through the viral oncoproteins E6 and E7 [5].

Despite the availability of preventive strategies and treatments such as platinum-based chemotherapy [6], disease progression and recurrence remain significant challenges. This ongoing difficulty underscores the necessity for a deeper molecular understanding.

Post-translational modifications (PTMs) regulate protein function and stability in cancer [7]. Among these, lysine acetylation and ubiquitination are highly dynamic, interconnected, and often share target residues; however, they are usually studied separately, leaving their joint regulation unclear. This gap raises the question of how their interplay shapes proteome regulation in cancer, a relationship that remains poorly understood. Both modifications are central to protein stability, DNA repair, and apoptosis processes notably affected by HPV. Specifically, acetylation, regulated by KATs and KDACs, modulates chromatin dynamics and transcription [8,9], while ubiquitination, through E1–E2–E3 enzyme cascades [10], targets proteins for proteasomal degradation. Importantly, up to 30% of lysine sites may overlap for these modifications, creating potential for competition or cooperation [11].

We focused on these two modifications for several reasons. Both target the ε-amino group of lysine, so they cannot occupy the same residue of a single molecule at the same time. Both are tied to processes altered by HPV: E6 uses the ubiquitin ligase E6AP to degrade p53, and E6 and E7 interact with the acetyltransferases CBP/p300 and PCAF [12,13,14,15,16]. Both can also be quantified as site occupancy with the same chemical-labeling workflow [9,17,18]. Other lysine modifications, such as methylation, SUMOylation and NEDDylation, also compete for these residues [19] but were outside the scope of this study.

HPV exploits this molecular interplay: the E6 oncoprotein hijacks the ubiquitin ligase E6AP to induce p53 degradation [12], while E7 drives ubiquitin-mediated degradation of IFI16 to help the virus evade immune recognition [20]. Acetylation stabilizes p53 and protects it from ubiquitination; however, this regulatory balance is disrupted during infection [21].

High-resolution mass spectrometry now enables precise mapping and quantification of PTM occupancy [22], which enabling the study of regulatory networks. Evidence shows that acetylation and ubiquitination interact, affecting protein stability, localization, and activity. However, their interplay at proteome-wide and site-specific levels, especially in cervical cancer, remains largely uncharacterized, leaving the roles and interactions of these PTMs in cervical cancer underexplored.

In this context an integrative, proteome-wide approach was used to map and quantify lysine acetylation and ubiquitination in human cervical cancer cells. The data show that both modifications can be quantified on the same lysine residues and that their occupancy differs among the cell models. These differences are particularly prominent in proteins involved in cell-cycle control, chromatin organization, and metabolic regulation. Collectively, these findings suggest that crosstalk between acetylation and ubiquitination may be an underappreciated regulatory layer in cervical cancer cells, and they point to candidate epigenetic and proteostatic vulnerabilities that remain to be tested.

Beyond describing acetylation and ubiquitination in isolation, this study frames the ubiquitin-proteasome system (UPS) as a pleiotropic regulator of cellular homeostasis. Using a site-specific, stoichiometric map of ubiquitination across four cervical cell models with distinct HPV/p53 backgrounds (SiHa, HeLa, C33A, and HaCaT), we compare ubiquitination with lysine acetylation at shared residues. The HPV-negative C33A line showed a larger ubiquitinome, which could reflect compensatory proteostatic surveillance in the absence of viral E6/E7 activity. These established cell lines, however, differ in more than HPV status (histology, and TP53 status and genetic background), and they do not reproduce the heterogeneity of primary tumors, so the differences reported here describe these models and cannot be attributed to HPV alone. This interpretation would extend UPS regulation beyond simple protein turnover to intersect transcriptional regulation, genome maintenance, antiviral signaling and metabolic control, consistent with its pleiotropic role in homeostasis.

## 2. Results and Discussion

### 2.1. Comprehensive PTM Profiling Reveals Cell Line-Specific Acetylation and Ubiquitination Landscapes

**Figure 1:**
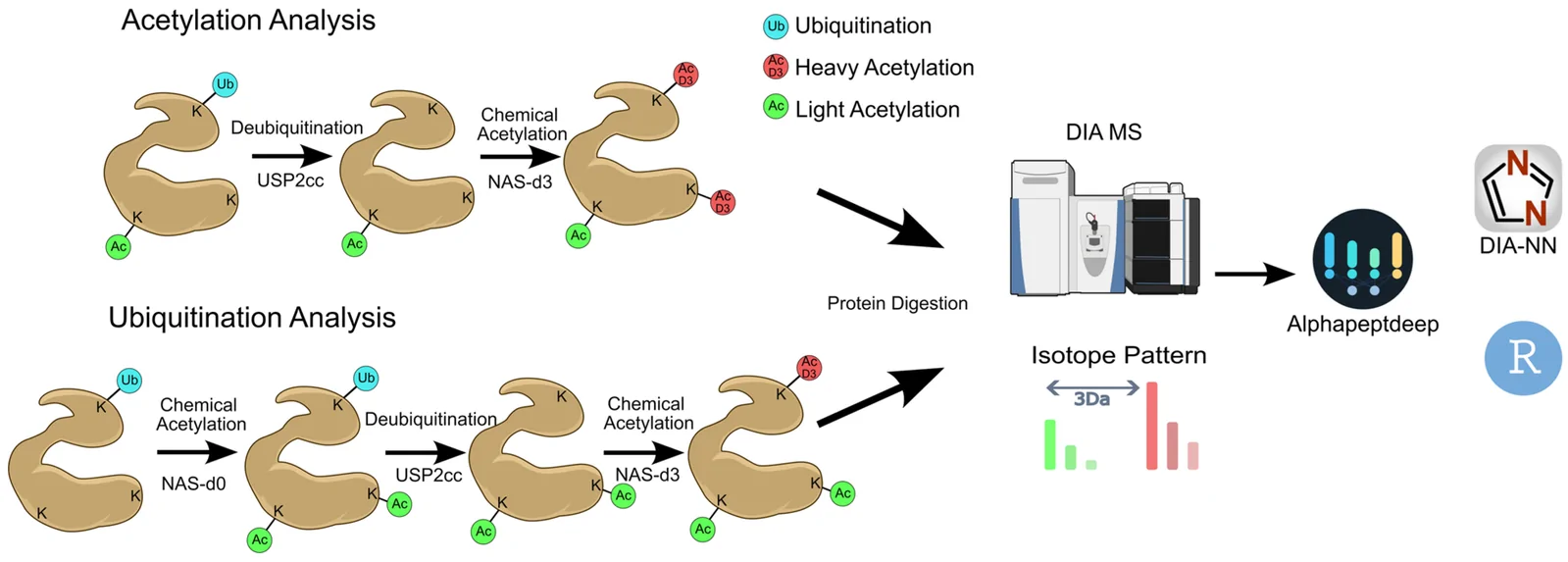
Graphical representation of the workflow for identifying and quantifying protein acetylation and ubiquitination using chemical acetylation and mass spectrometry, followed by bioinformatics analysis. Created with Inkscape 1.4 (https://inkscape.org) using illustrations from NIH NIAID BioArt (https://bioart.niaid.nih.gov).

A DIA-MS-based stoichiometry quantification approach using chemical labeling to tag lysine residues was employed to characterize the PTM landscape in cervical cancer (Figure 1). Four cell lines with distinct molecular backgrounds were analyzed: SiHa (HPV16-positive), HeLa (HPV18-positive), C33A (HPV-negative, harboring the p53 R273C mutation), and HaCaT (immortalized keratinocyte control). Because these lines differ in terms of HPV status, TP53 status, histology and genetic background, this model does not allow the individual contributions of viral oncoproteins and p53 mutation to be separated.

Each cell line was analyzed in three biological replicates, and the acetylation and ubiquitination workflows used separate cell pellets from the same cultures. No PTM enrichment was performed. In the acetylation workflow, ubiquitin was first removed with USP2cc and all free lysines were labeled with NAS-d3, so the light channel (d0) corresponds to pre-existing acetylation. In the ubiquitination workflow, free lysines were first blocked with NAS-d0, so the light channel contains both pre-existing acetylation and chemically acetylated lysines; lysines exposed after USP2cc treatment were then labeled with NAS-d3. Both forms of a peptide are measured in the same run, so the ratio refers to the measured pool of that peptide rather than to modified-peptide intensity alone.

**Figure 2:**
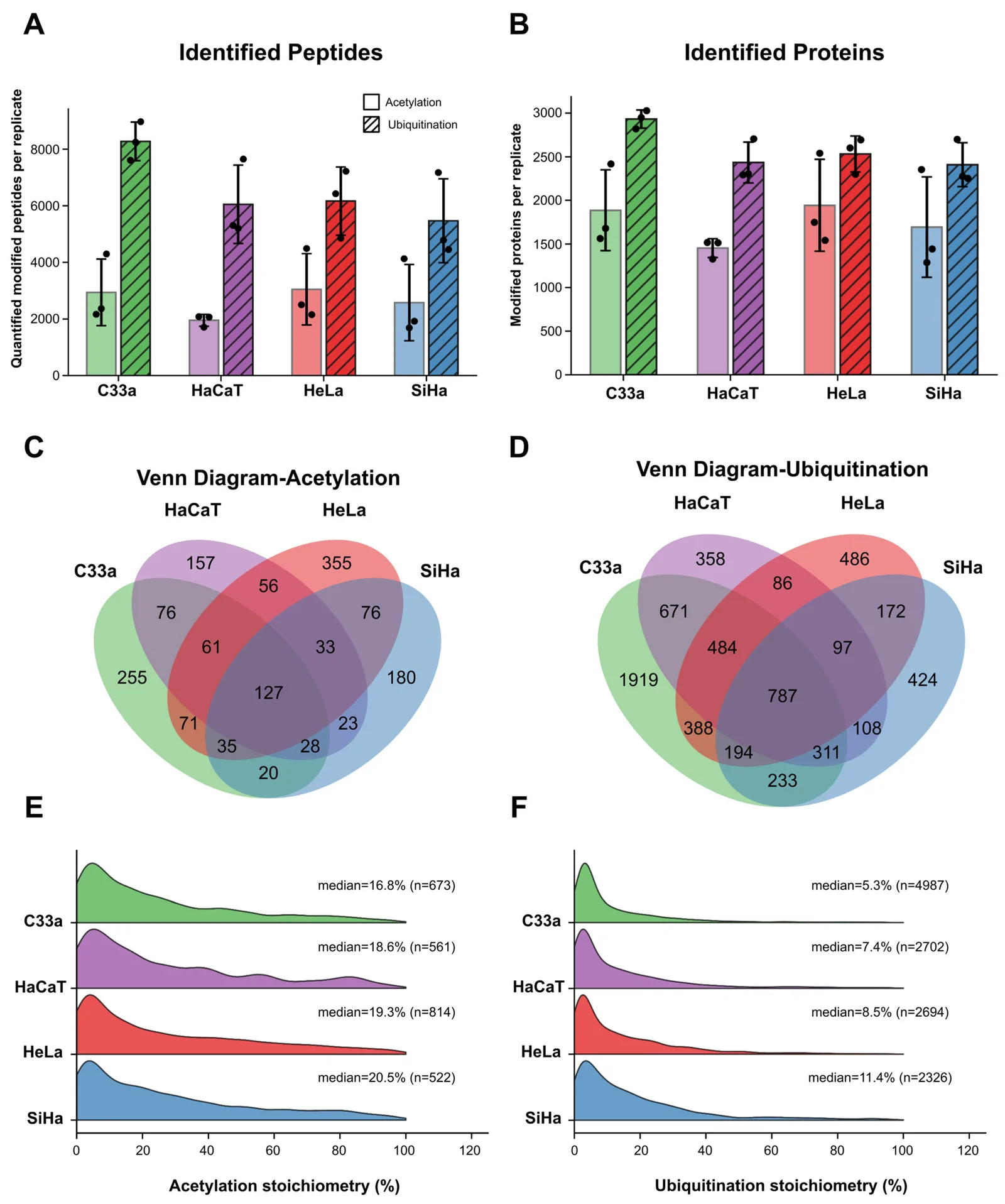
Identification of modified peptides and proteins and stoichiometry distributions in the four cell lines. (**A**) Number of acetylated and ubiquitination-associated peptides identified in C33A, HaCaT, HeLa and SiHa cells (solid bars, acetylation and hatched bars, ubiquitination). (**B**) Number of acetylated and ubiquitination-associated proteins identified in each cell line. In (**A**,**B**), bars show the mean ± SD of three biological replicates and points show the individual replicates. Values are identification counts and were not tested statistically. (**C**) Venn diagram of acetylated peptides quantified in all three biological replicates of each cell line (1553 peptides in total). (**D**) Venn diagram of ubiquitination-associated peptides quantified in all three biological replicates of each cell line (6718 peptides in total). Peptide lists for each region are provided in Supplementary Tables S1 and S2. (**E**) Ridgeline plot of acetylation stoichiometry in each cell line. (**F**) Ridgeline plot of ubiquitination stoichiometry in each cell line. In (**E**,**F**), each distribution shows the mean stoichiometry of the peptides quantified in all three replicates of that line. The medians were 16.8% (C33A), 18.6% (HaCaT), 19.3% (HeLa) and 20.5% (SiHa) for acetylation, and 5.3%, 7.4%, 8.5% and 11.4%, respectively, for ubiquitination.

Comparative analysis showed differences in lysine-centered PTM landscapes among the cervical cell models (Table 1) (Figure 2A,B). Notably, overall proteome coverage was highly consistent across all four cell lines, with total peptide identifications per replicate in the acetylome analysis ranging from 37,638 to 40,509 and total protein identifications ranging from 6648 to 6799 (means of three replicates), reflecting less than 8% and 3% variation, respectively. In the ubiquitinome analysis, total peptide identifications ranged from 31,860 to 35,048, and total protein identifications ranged from 6091 to 6383, with variations of 10% and 5%, respectively. This comparable depth of proteome sampling argues against gross differences in sampling depth, although modification-specific detection biases cannot be excluded. The counts below are identification counts and were not tested statistically. In contrast, PTM-specific landscapes showed marked divergence. The HPV-negative C33A cell line showed a larger ubiquitinome, with 4987 peptides across 2043 proteins quantified in all three replicates, a 1.9–2.1-fold increase compared to HPV-positive lines (per replicate, 8275 ± 681 versus 5471–6166 peptides in the other lines). This expanded ubiquitination profile could reflect compensatory adaptation of proteostasis networks in the absence of viral E6/E7-mediated degradation pathways. In HPV-positive contexts, these viral proteins redirect the ubiquitin–proteasome system toward selective degradation of key tumor suppressors, including p53 and other cellular proteins for proteasomal degradation [12,23,24]. Conversely, HeLa cells showed the largest number of acetylated peptides, with 814 peptides across 704 proteins, which is consistent with increased CBP/p300 acetyltransferase activity in HPV-positive cervical cancer [13,14,15,25]. In line with this, p300 protein was detected only in SiHa and HeLa cells, although lack of detection in the other lines does not prove its absence. The observed divergence between acetylation and ubiquitination profiles across cell lines suggests that the balance between lysine-modification states differs among these models.

Values represent identification counts, not quantitative measurements; no statistical comparison was performed on these data. Total peptides and proteins are the mean ± SD of three biological replicates; modified peptides and proteins are those quantified in all three replicates (per-replicate counts in Table S5). Statistical analysis (one-way ANOVA, FDR-corrected, with Tukey post hoc tests) was applied to peptide-level stoichiometry values only, as described in the data analysis subsection.

Venn diagram analyses of the acetylated and ubiquitinated peptides (Figure 2C,D) revealed asymmetric distributions of shared and unique PTMs, supporting the existence of differential lysine-modification hierarchies. HeLa exhibited the highest number of unique acetylated peptides with 355, followed by C33A (255), SiHa (180), and HaCaT (157). For ubiquitination, C33A exhibited the greatest number of unique ubiquitinated peptides (1919), followed by HeLa (486), SiHa (424), and HaCaT (358).

The strong asymmetry between acetylation and ubiquitination may suggest competition for overlapping lysine residues, which may influence protein fate decisions through mutually exclusive or sequential modification states. However, it is important to acknowledge that technical and stochastic factors may also contribute to the observed asymmetry. These include differences in the number of proteins identified per condition, the inherently low stoichiometry of PTM sites, stochastic peptide sampling during data acquisition, and suboptimal quantification of low-abundance modified species. Therefore, the biological interpretation of lysine crosstalk should be considered in the context of these methodological limitations. A mechanistic model is proposed in which HPV-positive cervical cancer cells employ viral E6/E7 oncoproteins to bias lysine-modification equilibria toward selective ubiquitin-mediated degradation, while concurrently enhancing acetylation-dependent transcriptional plasticity [26,27]. In contrast, HPV-negative C33A cells may compensate for the absence of viral regulatory mechanisms by expanding endogenous ubiquitin-dependent quality control pathways, potentially mitigating proteotoxic stress caused by accumulated mutant p53. In this framework, lysine residues may function as regulatory hubs where acetylation and ubiquitination dynamically compete or cooperate, influencing protein stability, functional state, and signaling output.

### 2.2. Stoichiometry Distributions Suggest Distinct Regulatory Architectures for Lysine Acetylation and Ubiquitination

A central objective of this study was to quantify site-specific PTM stoichiometry using a DIA-MS workflow, thereby enabling direct comparison of regulatory occupancy across acetylation and ubiquitination landscapes. Stoichiometry distributions differed between the two modifications (Figure 2E,F). Only peptides quantified in all three biological replicates of a cell line were considered for that line. Median replicate coefficients of variation were 20–32% for acetylation and 29–38% for ubiquitination. Labeling and deubiquitination efficiencies were not measured experimentally, and no correction factor was applied; as an indirect indicator, only 0.08% of identified peptides ended in lysine (versus 98.6% in arginine), which is consistent with near-complete lysine acetylation.

Acetylation stoichiometry displayed a broad distribution, with substantial numbers of sites showing intermediate to high occupancy and medians of 16.8–20.5% across cell lines (Figure 2E). This distribution contrasts sharply with previous reports of extremely low global acetylation stoichiometry of only 0.02% in HeLa cells measured by SILAC-based approaches [28]. The stoichiometry values reported here are notably higher than those in the broader literature, and the discrepancy warrants careful consideration. No PTM enrichment was performed in this workflow, so the difference cannot be attributed to enrichment; technical factors, such as incomplete labeling, peptide recovery biases, interference in the low-abundance channel, and quantification accuracy at low-abundance sites, likely contribute to the apparent elevation. As such, absolute stoichiometry values should be interpreted with caution and primarily considered in relative, within-experiment comparisons rather than as direct measures of global acetylation occupancy. Notably, high stoichiometry acetylation sites were predominantly localized to nuclear proteins involved in transcriptional regulation and chromatin remodeling. This is consistent with models in which acetylation acts as a stable regulatory signal modulating protein function and transcriptional output, as well as with prior work demonstrating that high-occupancy acetylation preferentially occurs on transcriptional regulators, acetyltransferases, and histone substrates [28].

In contrast, ubiquitination exhibited a markedly different stoichiometric profile, characterized by low site occupancy, with median values of 5.3–11.4% across cell lines (Figure 2F). These values are also considerably higher than those reported in recent global analyses, which likely reflects the technical factors discussed above. However, although the lower occupancy relative to acetylation is consistent with analyses showing that ubiquitination occupancy spans several orders of magnitude and is typically substantially lower than phosphorylation occupancy (median 0.0081% for ubiquitination vs. 2.5% for phosphorylation) [29]. The low occupancy of ubiquitination site occupancy reflects the rapid-turnover kinetics inherent to ubiquitin-mediated signaling, in which approximately 67% of ubiquitylation sites exhibit half-lives below thirty minutes under basal conditions [30,31]. Such low steady-state occupancy enables highly dynamic signaling while conserving the finite cellular ubiquitin pool (approximately 4.5 × 10^7^ molecules per HeLa cell) [29], suggesting that ubiquitination functions as a transient regulatory mark optimized for rapid proteostatic decision-making.

Together, these findings are consistent with a model in which lysine acetylation and ubiquitination occupy distinct regions of regulatory space: acetylation primarily operates as a stable regulatory modification that alters protein activity, protein–protein interactions, and subcellular localization [8,32]. The higher occupancy observed for acetylation sites (10–50% range) is consistent with modifications that must be present at sufficient levels to exert direct effects on protein function in the cell. In comparison, ubiquitination acts as a low-occupancy, high-flux signal enabling cells to maintain regulatory flexibility while conserving ubiquitin pools [29]. Within this framework, lysine residues may act as competitive regulatory nodes where differential stoichiometry could influence whether proteins adopt stable functional states or enter degradation and quality-control pathways.

To examine whether regulatory enzyme abundance predicts acetylation stoichiometry, we performed Spearman rank correlations between the protein levels of 16 KATs and KDACs and the run-level median acetylation stoichiometry across the 12 biological samples (Figure S1A, Table S6). No enzyme showed a significant correlation after Benjamini–Hochberg correction; the strongest nominal trends were for ELP3 (ρ = −0.36 and *p* = 0.26) and HAT1 (ρ = −0.31 and *p* = 0.33), and these were not interpreted further. A precedent for this type of correlation between enzyme abundance and stoichiometry correlation exists in human cervical cancer-cell lines: Gil et al. (2017) reported that higher SIRT1 abundance correlates with lower acetylation occupancy across HeLa, SiHa and CaLo cells [9]. This lack of association is consistent with the broader observation that most lysine acetylation occurs at very low stoichiometry and that class I deacetylases preferentially target the subset of high-occupancy sites, while writer abundance has comparatively little predictive power over site-level acetyl occupancy [28,33].

A parallel analysis for ubiquitination included 97 E3 ligases and DUBs (Figure S1B, Table S6). No enzyme reached statistical significance after correction; the strongest nominal associations (USP28, ρ = −0.78, *p* = 0.003; and RNF123, ρ = +0.73, *p* = 0.007) had FDR values ≥ 0.19 and are not interpreted as evidence of regulation. The overall absence of significant correlations between enzyme abundance and ubiquitination stoichiometry across the four cell lines is consistent with the known architecture of the ubiquitin system: humans encode > 600 E3 ligases and ~100 DUBs [34], and substrate modification depends far more on enzyme activity than on abundance. This point is reinforced by the finding that USP7 inhibition reshapes the ubiquitinome within minutes without changes in DUB protein levels [35], and by direct proteome-wide evidence showing no correlation between protein abundance changes and ubiquitination-site dynamics [36]. Beyond substrate redundancy, E3 ligase activity is regulated post-translationally by autoinhibition, cullin neddylation, and substrate clustering [37], all of which decouple enzyme concentration from modification output.

### 2.3. The Global PTM-Landscape Organization Distinguishes HPV-Positive and HPV-Negative Regulatory States

**Figure 3:**
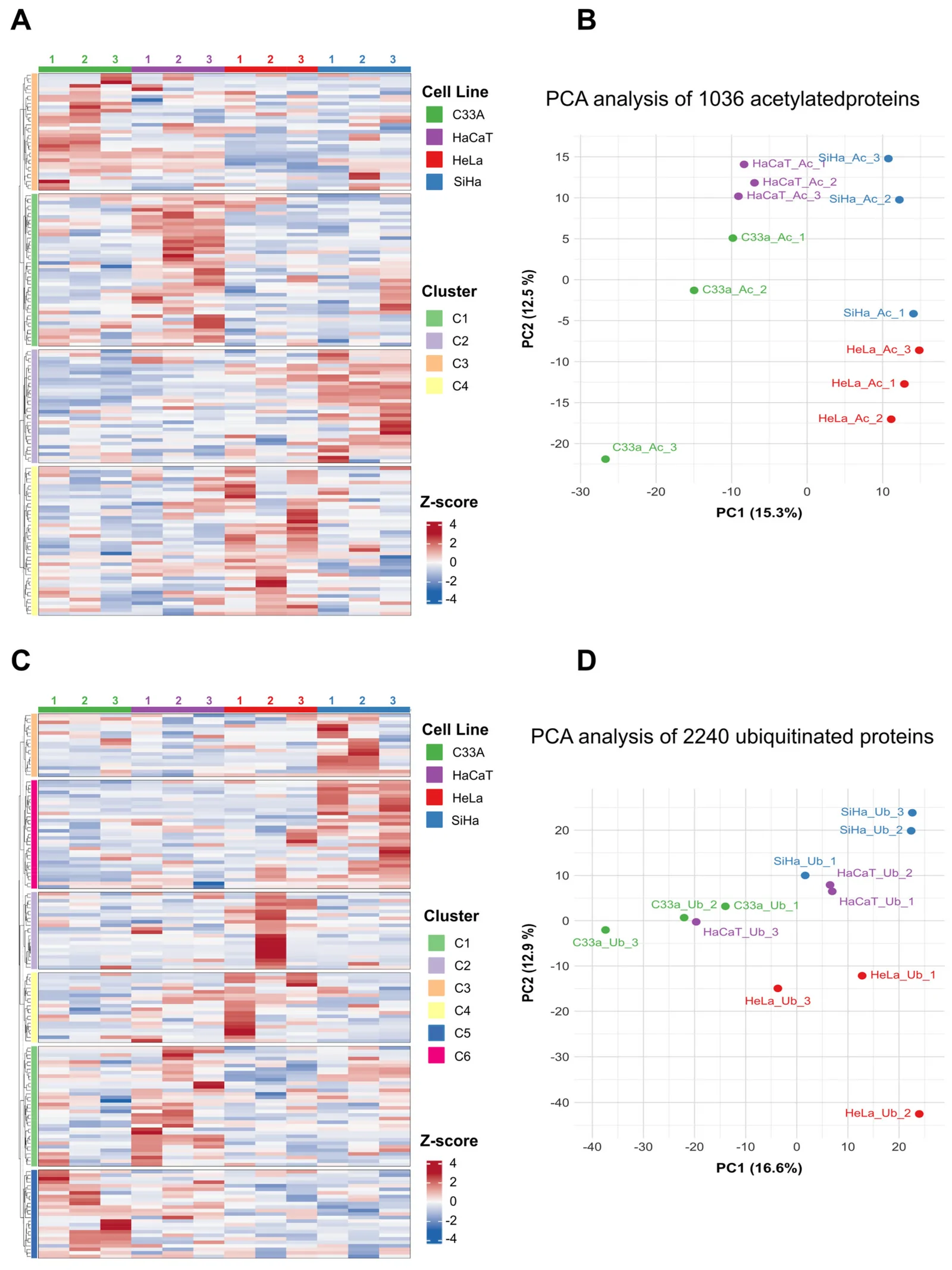
Hierarchical clustering heatmaps and PCA of the acetylated and ubiquitinated proteins. (**A**) Hierarchical clustering heatmap of the 150 most variable acetylated proteins across the C33A, HaCaT, HeLa and SiHa cell lines. (**B**) PCA analysis of 1036 acetylated proteins across the four cell lines. (**C**) Hierarchical clustering heatmap of the 150 most variable ubiquitinated proteins across the four cell lines. (**D**) PCA analysis of 2240 ubiquitinated proteins across the four cell lines.

To evaluate higher-order organization of PTM landscapes, we performed hierarchical clustering and principal-component analysis (PCA) of quantified acetylated and ubiquitinated proteins (Figure 3).

Heatmap analysis of the 150 most variable acetylated proteins revealed distinct clustering patterns that separated HPV-positive cell lines from HPV-negative C33A cells and the HaCaT control (Figure 3A) (Table S1). Remarkably, proteins involved in chromatin remodeling complexes and transcriptional regulation contributed substantially to this separation, including components of the CBP/p300 acetyltransferase machinery and their downstream targets. This finding aligns with established mechanisms in which HPV E6 and E7 oncoproteins modulate CBP/p300 activity, thereby reshaping host transcriptional programs through altered acetylation patterns and gene expression dynamics [16,38]. C33A’s separation was driven in part by a distinct set of epigenetic regulators absent from the HPV-positive line, such as UHRF1, a multidomain reader that coordinates DNA methylation with histone modification status, and KDM1A (LSD1), whose acetylation state directly modulates its H3K4 demethylase activity, pointing to a p53 mutation-driven epigenetic reprogramming axis that operates independently of viral oncoproteins [39,40].

PCA analysis of 1036 acetylated proteins further demonstrated that HPV-positive cell lines clustered closely along the primary principal component, whereas C33A and HaCaT occupied distinct regions of PTM space (Figure 3B). Loading analysis identified proteins involved in RNA processing, ribosomal biogenesis, and mRNA splicing as major contributors to separation along PC1, in accordance with the known roles of HPV oncoproteins (E6/E7) in reprogramming transcriptional and RNA-processing pathways [5,41]. Sub clustering within the HPV-positive group further distinguished SiHa from HeLa: SiHa carried an acetylation signature enriched for DNA repair factors, XRCC1, RAD50, and ARID2, while HeLa showed a profile centered on nuclear transport machinery and the DEK oncogene, consistent with HPV18 having a higher integrated viral copy number and broader disruption of host chromatin architecture [42,43]. These analyses suggest that viral oncogene expression represents a dominant organizing principle of the acetylome.

In contrast, ubiquitination landscapes exhibited a distinct clustering architecture (Figure 3C) (Table S2). The 150 most variable ubiquitinated proteins segregated C33A into a unique cluster distinct from those of all other cell lines, reflecting the expanded ubiquitinome observed in C33A cells and suggesting that p53 mutation-associated alterations fundamentally reshape ubiquitin-dependent proteostatic networks. Within this cluster, SIRT6, a NAD^+^-dependent deacetylase that promotes NHEJ-mediated DSB repair and base excision repair, and regulates telomere integrity through TRF2 deacetylation, acts as a central genome maintenance factor, occupying the regulatory niche that E6/E6AP complexes fill in HPV-positive lines [44]. Its co-occurrence with KRT5 and KRT14, basal-layer keratins absent from all HPV-positive lines, indicates that C33A retains squamous differentiation identity while relying on intrinsic chromatin guardians rather than viral cooperative mechanisms to sustain transformation. Ribonucleotide reductase RRM1 and nucleoside monophosphate kinase CMPK1 were also C33A-specific contributors, suggesting that dNTP pool regulation, rather than replication-fork protection or homologous recombination, is C33A’s primary replication stress-management strategy [45].

PCA of 2240 ubiquitinated proteins reinforced this observation, with C33A occupying a clearly separated position within PTM space (Figure 3D). Loading analysis identified proteasomal components, ubiquitin-conjugating enzymes, and metabolic enzymes as major contributors to cell-line discrimination, suggesting large-scale remodeling of proteostasis networks in the absence of viral regulatory inputs. The E3 ligase repertoires contributing to HPV-positive cell-line positioning were serotype-specific: HeLa carried UBE3C, HERC4, and RNF213, while SiHa relied on UBE4B, NSMCE1, and CDC2. These distinct substrate-targeting programs are shaped by HPV18 versus HPV16 oncoproteins, respectively [42,46].

Interestingly, despite clear differences in acetylation profiles, HeLa and SiHa exhibited relatively similar ubiquitination landscapes, suggesting convergence of HPV16- and HPV18-driven ubiquitin signaling toward shared substrate networks. That convergence likely operates primarily through E6/E6AP-mediated p53 destruction, a substrate common to both HPV16 and HPV18 E6 proteins, while the broader immune-evasion programs diverged: HeLa specifically deployed HERC4 to degrade STING and UBE3C to target IRF3/7, an innate immune suppression axis with no equivalent in SiHa [46,47]. These observations imply that acetylation and ubiquitination provide complementary, partially orthogonal layers of molecular classification: whereas the acetylome captures the transcriptional reprogramming associated with viral oncogenesis, the ubiquitinome reflects proteostatic adaptation and quality-control dynamics.

### 2.4. Differential Stoichiometry Identifies PTM-Dependent Regulatory Nodes

**Figure 4:**
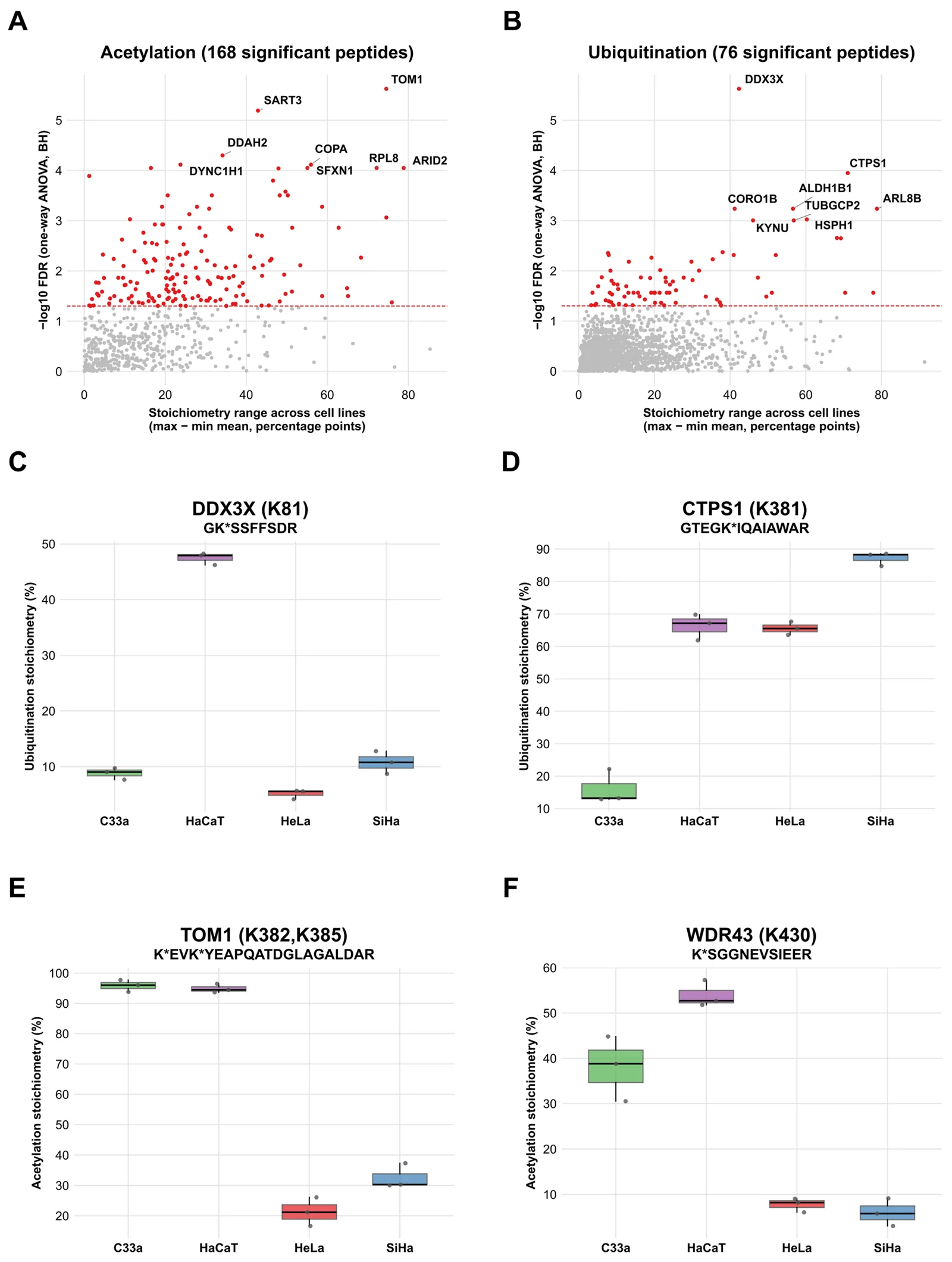
One-way ANOVA (across the four cell lines: C33A, HaCaT, HeLa and SiHa) with Benjamini–Hochberg FDR correction (adjusted *p* < 0.05) applied to peptide-level stoichiometry values. (**A**) ANOVA results for acetylated peptides (−log10 FDR versus the range of mean stoichiometry across cell lines). (**B**) ANOVA results for ubiquitinated peptides. In (**A**,**B**), red points are significant peptides (FDR < 0.05), grey points are non-significant, the dashed line marks FDR = 0.05, and the eight peptides with the lowest FDR are labeled. (**C**) Ubiquitination stoichiometry of DDX3X K81. (**D**) Ubiquitination stoichiometry of CTPS1 K381. (**E**) Acetylation stoichiometry of TOM1 K382/K385. (**F**) Acetylation stoichiometry of WDR43 K430. In (**C**–**F**), grey dots are biological replicates and boxes show the median and interquartile range. K* marks the quantified lysine; because all lysines are chemically acetylated in both workflows, the formerly ubiquitinated lysine is detected as acetyl-d3. Pairwise Tukey results are in Table S3.

To identify proteins with significant cell line-specific differences in terms of modification levels, we performed one-way ANOVA with Benjamini–Hochberg FDR correction (*p* < 0.05) on each acetylated and ubiquitinated peptide quantified in all three replicates of at least two cell lines, followed by Tukey post hoc tests. This analysis identified 168 differentially acetylated peptides (160 proteins) and 76 differentially ubiquitinated peptides (75 proteins) (Figure 4A,B and Tables S1–S3). Of the 860 pairwise post hoc comparisons for these peptides, 550 were significant.

Although the absolute expansion of the ubiquitinome was more pronounced, the greater number of significantly differential acetylated peptides suggests that acetylation changes may be more consistently distributed across the proteome. Among the 59 differentially acetylated peptides quantified in all four lines, 13 had lower and 21 had higher acetylation in both HPV-positive lines than in both HPV-negative lines, whereas most ubiquitination differences were specific to a single cell line.

Collectively, these results are consistent with a model in which acetylation provides a relatively stable regulatory framework that defines cell state, whereas ubiquitination functions as a dynamic amplifier of proteostatic stress responses. Within lysine-centered regulatory networks, differential stoichiometry may influence whether acetylation stabilizes protein function or ubiquitination directs rapid turnover, a possibility that remains to be tested experimentally.

### 2.5. Important Differentially Ubiquitinated Proteins May Define Regulatory Axes Associated with Cervical Cancer-Cell Identity

Analysis of proteins exhibiting extreme ubiquitination variance identified a subset of candidates that may function as molecular signatures distinguishing the cell models.

Among these, the RNA helicase DDX3X showed the most significant difference across the dataset (K81 and FDR = 2.4 × 10^−6^), with HaCaT cells exhibiting the highest ubiquitination stoichiometry (47%) compared with 5–11% in the three cancer lines (Figure 4C). DDX3X participates in translation initiation and innate immune signaling, and differences in its ubiquitination may relate to the regulation of these processes in a context-dependent manner. CTP synthase 1 (CTPS1; K381; and FDR = 1.1 × 10^−4^) (Figure 4D), the rate-limiting enzyme of CTP synthesis, displayed cell line-specific ubiquitination patterns, with the lowest stoichiometry in C33A (16%) and the highest in SiHa (87%). Because nucleotide synthesis supports proliferation, these findings raise the possibility that ubiquitination may modulate metabolic entry points in a cell line-specific manner. Aldehyde dehydrogenase 1B1 (ALDH1B1 and FDR = 5.8 × 10^−4^) showed elevated ubiquitination in HeLa cells, and the lysosomal small GTPase ARL8B (FDR = 5.8 × 10^−4^) showed lower ubiquitination in SiHa. Whether these differences affect the turnover, localization or activity of the corresponding proteins remains to be tested.

Additional proteins with significant differences in ubiquitination included MORF4L2 (K116), a chromatin remodeling factor; HSPH1 (K44), a heat-shock chaperone; SEPTIN8 (K331), a cytoskeletal protein; and DYNC1H1 (K2399/K2401), the cytoplasmic dynein heavy chain. Replicate-level data for other candidate proteins (CST1, HEXA, GFPT1, FABP5, CLPTM1, HBS1L and CCNC), which did not reach significance in the peptide-level analysis, are shown in Figure S3. The presence of chromatin regulators among these sites is consistent with the idea that ubiquitination participates not only in protein degradation but also in transcriptional and epigenetic regulation.

### 2.6. Proteostasis–Epigenetics Coupling Emerges Through Ubiquitination Hotspots

Because every lysine is chemically labeled in both workflows, acetylation and ubiquitination stoichiometry can be compared at the same residue. We matched both datasets by peptide sequence and counted a lysine as shared only when it was the only lysine in the peptide, since peptides with several lysines cannot be assigned to an individual residue. In total, 553 single-lysine peptides from 470 proteins were quantified in all three replicates of at least one line in both workflows; 414 of these were quantified in the same line (Table S4); another 121 multi-lysine peptides are listed separately and were not counted. At these shared lysines, the sum of acetylation and ubiquitination stoichiometry did not exceed 100% in 98.5% of site–line pairs (median 25.7%), indicating that unmodified, acetylated and ubiquitinated forms of the same lysine coexist in the cell population. Because the two workflows used separate pellets, the values describe the population of molecules in each state, not individual molecules.

Only two shared lysines (TST K24 and NDUFS1 K289) differed significantly between cell lines in both workflows. More often, the two modifications differed at different lysines of the same protein, as in CTPS1 (acetylation at K466 and ubiquitination at K381) and DYNC1H1. We refer to this as protein-level overlap, which does not by itself indicate competition at a common residue. Given that acetyl-CoA availability, which is directly tied to metabolic state, is a primary determinant of acetylation capacity [48], ubiquitination-mediated control of metabolic enzymes such as CTPS1 could indirectly reshape the acetylation landscape [49,50]. These shared lysines are candidates for perturbation experiments testing direct competition.

### 2.7. Stoichiometry May Define Functional Dominance in Lysine PTM Competition

A central and often underappreciated dimension of PTM crosstalk is not simply which modifications occur, but at what occupancy. Our quantitative data suggest that acetylation and ubiquitination occupy different regions of stoichiometric space, although the two workflows are subject to different technical biases: acetylation exhibited intermediate to high site occupancy—consistent with its role in stabilizing protein conformation and maintaining transcriptionally active chromatin states—while ubiquitination operated at predominantly low occupancy with high variability across cell lines, which is consistent with the rapid-turnover kinetics inherent to proteostatic signaling.

This stoichiometric asymmetry may have functional consequences. At shared lysine residues, the relative balance between modifications, rather than their mere presence, could influence regulatory outcome. Mechanistically, sustained acetylation may limit ubiquitin conjugation through steric occlusion or competition for the same lysine, while even low-level ubiquitination can direct proteins toward degradation when acetylation is reduced or absent. In our dataset, this balance appears to differ between models: HPV-positive cell lines shared differences in acetylation of transcriptional regulators, whereas the HPV-negative C33A line showed a larger ubiquitinome. These differences suggest that the quantitative balance between competing marks may vary with HPV status, although other differences between the lines could also contribute, and direct competition was not tested.

### 2.8. Differential Acetylation Targets Converge on Metabolic, Immune, and Transcriptional Regulatory Axes

Among the most striking examples, sulfide:quinone oxidoreductase (SQOR; K65, FDR = 9.0 × 10^−5^) showed differential acetylation, with lower stoichiometry in HeLa (1.4%) than in C33A (17.9%) and HaCaT (10.9%). Given SQOR’s central role in mitochondrial sulfide metabolism and redox control, acetylation may stabilize mitochondrial proteins, which could modulate cellular redox state [51]. Interferon-induced protein with tetratricopeptide repeats 3 (IFIT3, F = 117.85) (Figure 4E) provides a complementary perspective on how acetylation dynamics relate to immune modulation. Elevated acetylation in HPV-negative or less oncogenically driven contexts suggests that acetylation may preserve antiviral signaling components by limiting ubiquitin-dependent turnover [52]. Conversely, reduced acetylation in HPV-positive models may facilitate ubiquitination-driven suppression of innate immunity, with differential acetylation potentially regulating antiviral activities. IFIT3 acetylation was lower in HeLa (6%) than in SiHa (35%); whether this relates to differences between HPV18 and HPV16 cannot be determined in these cell lines. Similarly, acetylation of Target of Myb1 membrane trafficking protein (TOM1; K382/K385, FDR = 2.4 × 10^−6^; Figure 4E), showed acetylation stoichiometry of about 95% in C33A and HaCaT versus 21–33% in HeLa and SiHa. An adaptor involved in endosomal sorting of ubiquitinated cargo, TOM1 highlights a regulatory intersection between trafficking and proteostasis [53]. We propose that acetylation of TOM1 may influence its ability to bind ubiquitinated substrates or membrane compartments, thereby modulating vesicular sorting pathways that are essential for viral entry, intracellular trafficking, and receptor recycling. If confirmed, such regulation would suggest that acetylation does not simply oppose ubiquitination but can modify ubiquitin signaling outcomes.

Acetylation changes observed in mediator complex subunit 11 (MED11; K103, FDR = 9.4 × 10^−4^) and poly(ADP-ribose) polymerase 9 (PARP9; K191, FDR = 5.3 × 10^−4^) additionally indicate that the lysine-modification switch extends to transcriptional regulation and immune signaling pathways. Increased acetylation of MED11 in HPV-positive lines (14% versus 3% in C33A) may stabilize mediator complex assembly [54], reinforcing transcriptional rewiring driven by viral oncogenes [55], while differential acetylation of PARP9 (HeLa and SiHa show higher acetylation, whereas C33A shows lower stoichiometry) suggests PTM-dependent modulation of interferon responses. Together, this evidence is consistent with a model in which acetylation selectively stabilizes regulatory hubs that define subtype-specific transcriptional and immune states.

Additional proteins with significant differences in acetylation included WDR43 (ribosome biogenesis; K430, FDR = 9.1 × 10^−5^; Figure 4D), UFD1 (ubiquitin recognition factor in ERAD; K42/K45, FDR = 1.6 × 10^−4^), ARID2 (SWI/SNF subunit; K15, FDR = 9.0 × 10^−5^), and ALG3 (N-glycosylation enzyme; K22, FDR = 8.7 × 10^−3^). WDR43 and UFD1 showed lower acetylation in both HPV-positive lines, whereas ARID2 acetylation was higher in HeLa (80%) than in C33A and HaCaT (10–16%); SCCPDH and NLN were not significant in the peptide-level analysis (Figure S3). Notably, the identification of acetylation on proteins directly involved in ubiquitin recognition and degradation, such as UFD1, raises the possibility of feedback loops in which acetylation regulates components of the ubiquitin machinery.

### 2.9. A Proposed Lysine-Fate Code for Proteostatic Decisions in Cervical Cancer

The patterns emerging from our multi-layered analysis suggest a coherent regulatory logic in which lysine residues serve as integration points for competing post-translational signals. Across the four cell lines examined, the stoichiometries of acetylation and ubiquitination differed in ways that tracked HPV status and p53 mutational background, although these factors cannot be separated from other differences between the lines. We propose that this balance constitutes a “lysine-fate code”: a modification-level logic that may influence whether a given protein is stabilized, functionally reprogrammed, or directed toward degradation. This concept aligns with emerging evidence that competition among PTMs at shared lysine residues encodes proteome-scale regulatory outcomes [19].

HPV status was associated with the main separation of the acetylome in our dataset (Figure 3B). HPV-positive cell lines shared differences in acetylation of transcriptional and chromatin-associated proteins, which is consistent with E6/E7-mediated engagement of host acetyltransferase networks such as CBP/p300. Notably, HPV-driven acetylation of conserved lysine residues has been shown to directly regulate viral and host transcriptional programs [56], and viral oncoproteins may exploit lysine-modification dynamics rather than simply disrupting them. In contrast, the HPV-negative C33A line exhibited a markedly expanded ubiquitinome enriched in proteostasis and ERAD pathways, which may reflect the compensatory activation of endogenous quality-control mechanisms in the absence of viral degradation machinery. We propose that these differences correspond to distinct functional states, a possibility that would require isogenic models to test.

What makes this system biologically meaningful is that it connects several apparently distinct processes through a common regulatory axis. Differentially modified proteins in our dataset span mitochondrial metabolism, innate immune signaling, and vesicular trafficking. Acetylation of enzymes such as SQOR may stabilize metabolic configurations amid oncogenic pressure, a role consistent with the established link between lysine acetylation and metabolic sensing [57]. Acetylation of IFIT3 appears linked to antiviral pathway maintenance, while TOM1 acetylation may influence ubiquitinated cargo sorting. If confirmed, the convergence of these functional domains on lysine regulatory hubs would suggest that PTM competition operates at the proteome scale rather than protein by protein. Targeting the enzymatic machinery that governs this regulation (acetyltransferases, deacetylases, E3 ligases, and deubiquitinases) could, therefore, be explored as a therapeutic strategy, although this remains to be tested.

### 2.10. Functionally Coherent Protein Networks and Lysine-Fate Regulation

**Figure 5:**
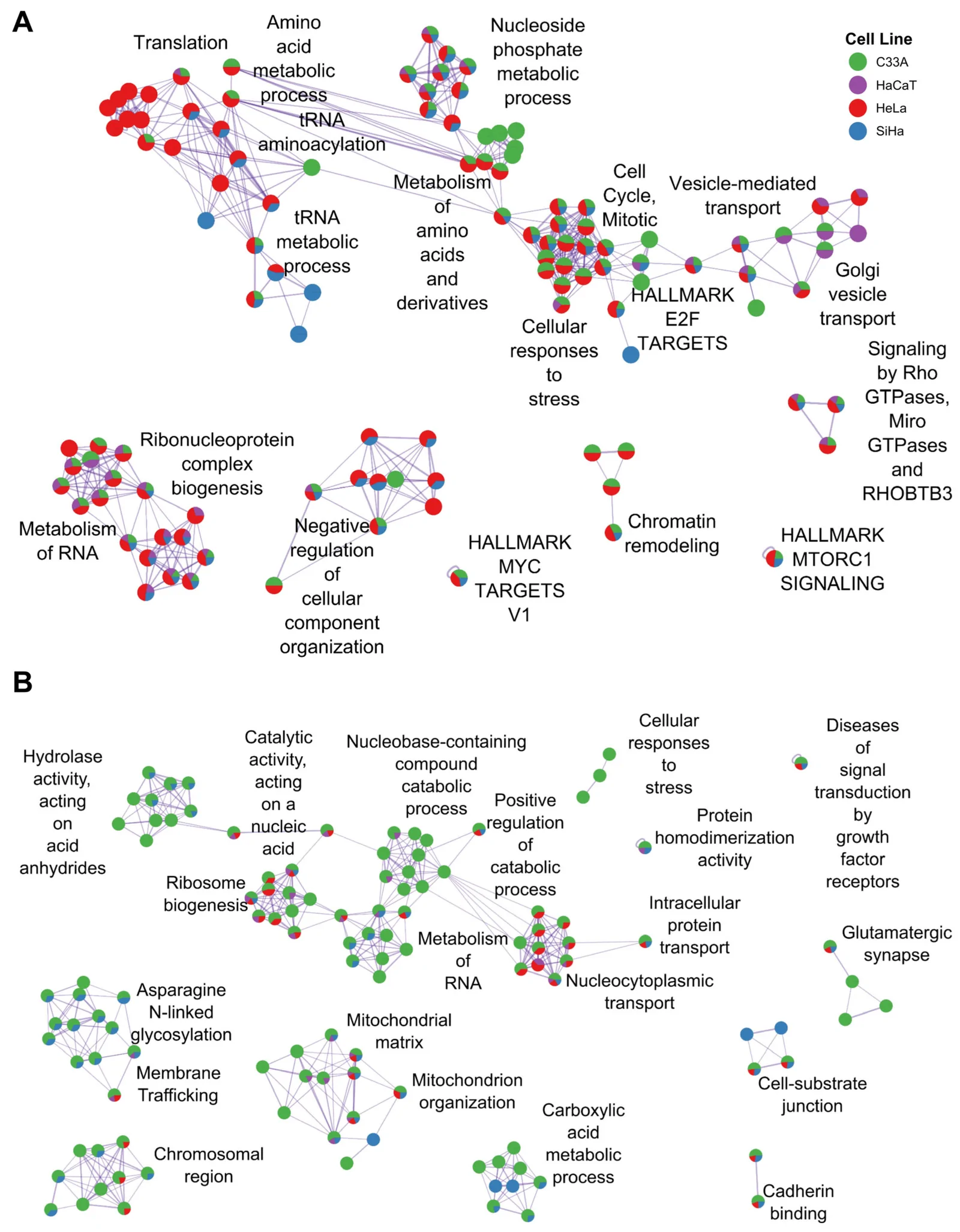
Pathway-enrichment analysis showing cell line-specific functional signatures. (**A**) Pathway enrichment of unique identified acetylated proteins for each cell line. (**B**) Pathway enrichment of uniquely identified ubiquitinated proteins for each cell line. Green denotes C33A, purple for HaCaT, red for HeLa, and blue for SiHa. The proportion of each circle that is filled represents the proportion of protein present in each cell line.

Functional-enrichment analysis of uniquely modified proteins grouped the proteins detected in a single cell line into functional modules. Because these proteins were defined by detection, the results describe the sets of identified proteins rather than biological exclusivity, and small modules are interpreted descriptively. Across cervical cancer-cell lines, distinct PTM-enriched pathways emerged that reflect divergent strategies for balancing transcriptional plasticity, proteostasis, metabolism, and immune regulation (Figure 5A,B).

In HPV16-positive SiHa cells, acetylated proteins were strongly enriched in RNA processing and mRNA splicing pathways (adjusted *p* < 0.001), while ubiquitinated proteins preferentially mapped to DNA repair and nucleotide excision repair processes. These findings may relate to the established mechanism by which HPV16 E6 drives E6AP-mediated degradation of p53 [58], which, in turn, reduces acetylation at key C-terminal regulatory lysines (K370, K372, K373, K381, K382, and K386). The resulting shift in lysine-modification balance may promote genomic adaptability by simultaneously dampening p53-dependent transcriptional programs and enhancing ubiquitin-dependent surveillance of DNA damage-response factors. p53 itself was not quantified in our data. This pattern could indicate that HPV16 oncogenesis reprograms lysine-modification networks to favor adaptive genome maintenance under persistent replicative stress.

HeLa cells exhibited a distinct regulatory architecture characterized by coordinated acetylation and ubiquitination within cell-cycle and mitotic-checkpoint pathways. Enrichment of chromatin-associated acetylation targets is consistent with a model in which HPV18 E7-driven recruitment of CBP/p300 reshapes transcriptional output [59].

Notably, acetylation of ribosomal proteins and translation-initiation factors (IFIT3, EIF4B, EIF4G1, RPS19, RPS3, RPL30, and RPL8) suggests that acetylation may regulate translational capacity at the systems level, extending PTM control beyond individual proteins to coordinated regulation of functional complexes. This observation is consistent with a model in which HPV18-positive cells may rely on acetylation-mediated stabilization of the biosynthetic machinery to sustain rapid proliferation.

In contrast, the HPV-negative C33A cell line showed a larger ubiquitinome enriched for protein-quality control, endoplasmic reticulum-associated degradation (ERAD), and proteostasis pathways (adjusted *p* < 0.001). The enrichment of ubiquitinated metabolic enzymes (GFPT1, FABP5, HEXA, ACAA1, ECHS1, and GATM), suggests metabolic reprogramming through ubiquitin-mediated enzyme turnover. The presence of ubiquitinated metabolic enzymes (e.g., ACAA1, ECHS1, and GATM) may indicate metabolic reprogramming through ubiquitin-mediated enzyme turnover, which would be consistent with the altered metabolism characteristic of cancer cells [60].

Acetylated proteins were enriched in metabolic pathways and mitochondrial functions, with SQOR and BCKDHA showing significant differences. The absence of viral E6-driven p53 degradation, combined with the R273C DNA contact mutation, creates a unique regulatory environment in which mutant p53 accumulates but lacks transcriptional activity, which may require alternative mechanisms to maintain proliferative capacity [61,62].

Several non-mutually exclusive hypotheses may explain the expanded ubiquitinome in C33A cells. (A) Compensation for absent E6-mediated degradation: in HPV-positive cells, E6/E6AP efficiently degrades p53 and other substrates, which may reduce the pool of proteins requiring ubiquitination through endogenous E3 ligases [63]. The absence of this viral degradation pathway in C33A may necessitate expanded endogenous ubiquitin-proteasome system activity. (B) p53 R273C-associated proteostatic stress: mutant p53 accumulation, combined with gain-of-function activities including enhanced cell proliferation and metabolic reprogramming, may impose proteostatic burdens requiring expanded ubiquitin-dependent quality control [64,65]. (C) Altered E3 ligase expression: the transcriptional programs controlled by wild-type p53, including regulation of MDM2 and other E3 ligases [21], are disrupted in C33A cells, potentially altering global ubiquitination machinery expression. These possibilities are consistent with a compensatory model in which ubiquitination becomes a dominant regulatory strategy when viral modulation of acetylation networks is absent. This model was not tested here because proteasome activity, protein half-life and E3/DUB activity were not measured.

The HaCaT keratinocyte control exhibited enrichment patterns associated with epithelial differentiation and cytoskeletal organization, establishing a baseline regulatory architecture from which oncogenic rewiring can be inferred. Compared with transformed lines, the HaCaT cells maintained PTM distributions consistent with their differentiated identities: keratins (KRT7 and KRT10), epithelial markers (FERMT2 and CALD1), and membrane trafficking proteins (LAMP1 and DAB2). These patterns areconsistent with the differences in lysine modification between transformed and non-transformed cells.

Comparative analysis of HPV16-positive (SiHa) and HPV18-positive (HeLa) cell lines revealed subtype-specific modulation of CBP/p300-associated acetylation networks. Although both E6 variants target the CBP/p300 acetyltransferase complex through interactions with the CH3 domain, inhibiting p53 acetylation at C-terminal regulatory lysines [15,63] and promoting ubiquitin-mediated degradation, quantitative differences in acetylation patterns suggest distinct mechanisms of acetylome remodeling between SiHa and HeLa cells. However, these lines also differ in terms of histology and genetic background.

HeLa and SiHa cells showed higher acetylation at several sites, including MED11 (mediator complex component) and PARP9 (poly(ADP-ribose) polymerase family member) (Tukey adjusted *p* < 0.001 versus C33A), and the chromatin regulator ARID2. This asymmetry is consistent with an effect of HPV E6/E7 oncoproteins on the acetylome, although it cannot be separated from other differences between the lines.

### 2.11. Acetylation–Ubiquitination Crosstalk and Lysine Fate

The coordinated patterns across cell lines are consistent with the possibility that acetylation and ubiquitination compete for the same lysine residues and that this competition influences protein fate [66,67]; our data do not test this directly.

At a shared lysine, the two modifications cannot occupy the same side chain of a single molecule at the same time. Acetylation tends to hold proteins in stable, active states; ubiquitination routes them toward degradation or signaling turnover. At the population level, however, the lysines quantified in both workflows carried unmodified, acetylated and ubiquitinated forms at the same time (Section 2.6), and these pools may differ between subcellular compartments and turnover states. The system is, therefore, better described as a dynamic distribution of modification states than as a binary switch. It is also not limited to two modifications: methylation, SUMOylation, NED-Dylation and other acylations compete for the same residues [19], and modifications elsewhere can act upstream, as N-terminal phosphorylation of p53 reduces MDM2 binding and, thereby, its C-terminal ubiquitination [68].

What complicates the picture is that the two machineries are not independent. Acetylation of UFD1, a ubiquitin recognition factor, could limit how efficiently the cell processes misfolded or surplus proteins. At the same time, SIAH1/SIAH2 E3 ligases ubiquitinate CBP/p300, TIP60, and PCAF, placing a ceiling on global acetylation output [69]. Each layer constrains the other. The cross-cell-line data are consistent with this feedback under distinct oncogenic conditions (HPV-positive lines, HPV-negative C33A, and the non-cancerous HaCaT line), although they do not demonstrate it.

HPV E6 disrupts the p53 methylation–acetylation–ubiquitination cascade by suppressing stabilizing acetylation while driving E6AP-dependent degradation. That mechanism is well documented for p53. Our data raise the possibility that similar rewiring extends to a broader set of substrates, since several proteins showed divergent acetylation and ubiquitination across the cell lines. Testing whether viral oncoproteins drive these changes will require isogenic models.

### 2.12. PTM Signatures and Systems-Level Regulatory States

Because stoichiometry is a ratio between isotope-coded forms of the same peptide, differences in occupancy are, assuming complete labeling, largely independent of protein abundance. TOM1 and WDR43 acetylation and DDX3X and CTPS1 ubiquitination are examples of cell-line-dependent modification patterns that expression-level proteomics would not detect. Lysine occupancy, and not only protein quantity, may, therefore, distinguish these cell models.

Our findings extend previous proteomic characterizations of cervical cancer cell lines and integrate with recent proteogenomic analyses of patient tumors [70,71]. A recent study identified EP300-driven acetylation of FOSL2-K222 as a cervical cancer proliferation driver [72]. FOSL2 was not among the top ANOVA hits, but MED11, ARID2, and GTF3C5—all part of the chromatin remodeling and general transcription machinery—did, each with significant cell-line-dependent acetylation differences. EP300 likely acetylates a much wider set of transcriptional substrates than FOSL2 alone, and which targets dominate depends on the oncogenic context.

IFIT3 and PARP9 stand out for a different reason. Both are interferon-stimulated genes, and both appear among the most differentially acetylated proteins across cell lines. The acetylation state of these innate immune proteins differs between HPV16 and HPV18 contexts. This finding could point to PTM-dependent mechanisms of immune modulationand might relate to the well-documented differences in immune clearance and oncogenic potential between the two viral subtypes [73]. However, HPV type cannot be separated from other differences between HeLa and SiHa.

The 168 acetylated and 76 ubiquitinated peptides with significant cell-line-dependent differences constitute a candidate pool for further study rather than a classifier. TOM1, WDR43, DDX3X, and CTPS1 are among the most significant. Whether these differences are reproducible in patient tumor specimens remains to be determined.

The data also suggest therapeutic hypotheses. C33A’s larger ubiquitinome could reflect greater proteasome dependence, which would be a testable vulnerability to proteasome inhibitors. HeLa’s acetylation profile suggests a potential role for HDAC inhibitors. Both ideas remain hypotheses that require drug-response and target-engagement experiments.

Collectively, the data describe a set of lysine sites whose occupancy differs among the cell models. We propose reflects a lysine-centered regulatory network in which HPV oncoproteins and other cell-line features shift the balance between stabilizing and degradative modifications to coordinate transcriptional and proteostatic programs simultaneously. This framework reframes PTM biology from isolated modification events toward a systems-level regulatory language in which modification stoichiometry may contribute to protein fate and cellular phenotype, a hypothesis that remains to be tested.

### 2.13. Methodological Considerations and Future Directions

This study demonstrates the utility of data-independent acquisition mass spectrometry (DIA-MS) for comprehensive quantification of acetylation and ubiquitination stoichiometry. Compared with traditional data-dependent acquisition (DDA) approaches, DIA provides greater reproducibility, fewer missing values, and broader dynamic range coverage [74,75].

No individual MS spectra are displayed in this manuscript. This study relies on large-scale, MS1-based quantification and site-level stoichiometry calculations rather than manual spectral interpretation; representative spectra were, therefore, not included as figures, which we note as a limitation. In addition, identifications rely on matching to predicted library spectra at 1% FDR, so a small proportion of identifications is expected to be incorrect, and predicted spectra do not provide independent confirmation of individual sequences.

However, several limitations should be considered. Cell-line models may not fully capture the heterogeneity of PTMs in patient tumors. Primary cervical-tumor specimens and patient-derived organoids will help validate findings in clinically relevant contexts. Second, steady-state stoichiometry analysis does not reveal the temporal dynamics of modification and turnover. Time-course experiments, including pulse-chase labeling or synchronized cell-cycle analysis with DIA-MS, would monitor PTM flux and turnover. Third, analysis of the whole lysates does not resolve subcellular PTM localization. Subcellular fractionation and organelle-specific enrichment are needed to study compartmentalized acetylation and ubiquitination. Fourth, not assessing ubiquitin-chain topology (K48 vs. K63 linkages) limits our understanding of degradation versus non-degradative outcomes [76]. Fifth, labeling and deubiquitination efficiencies were not measured, and the acetylation and ubiquitination workflows used separate pellets. Sixth, with only 3 Da between channels, the natural M+3 isotope of the light species can overlap the heavy channel, which may inflate absolute ubiquitination occupancy, particularly for larger single-lysine peptides; absolute values should, therefore, be interpreted with caution. Seventh, peptides with several lysines report only the fully light or fully heavy species and were not assigned to individual residues. Finally, sites were defined as lysines exposed by USP2cc rather than by K-ε-GG remnant detection, and a contribution of ubiquitin-like modifiers cannot be excluded without specificity controls.

Future work should also integrate additional PTMs, including phosphorylation, SUMOylation, and methylation, to better define PTM crosstalk networks. Functional validation, including CRISPR-based mutagenesis or modulation of CBP/p300 acetyltransferases and E3 ubiquitin ligases, will determine effects on stability, localization, and function. Key assays, such as protein turnover, transcriptional reported assays, and analyses of proliferation or apoptosis, will directly connect lysine PTM changes to phenotypes. Single-cell proteomics and targeted perturbation of acetyltransferases, deacetylases, ubiquitin ligases, and deubiquitinases could reveal whether lysine-fate decisions follow shared regulatory rules in cancer. Targeting enzymes that regulate the acetylation–ubiquitination balance may reprogram cancer states by shifting the balance between stability and degradation at lysine residues, a possibility that remains to be tested.

## 3. Materials and Methods

### 3.1. Cell Culture

The cell lines for this study were SiHa (derived from HPV 16-positive cervical squamous cell carcinoma), HeLa (derived from HPV 18-positive cervical adenocarcinoma), C33A (derived from HPV-negative cervical carcinoma), and HaCaT (a non-cancerous, immortalized cell line derived from human keratinocytes). The cell lines used in this study were obtained from Dr. Berenice Illades-Aguiar’s research group, which has previously employed these models in studies of cervical carcinogenesis and human papillomavirus-associated molecular alterations [77,78,79].

HaCaT cells (RRID:CVCL_0038) were obtained from Dr. Berenice Illades-Aguiar’s research group. The original commercial supplier could not be confirmed from the available records. Cellosaurus notes that CLS is the only HaCaT supplier officially approved by the German Cancer Research Center, although the line is distributed through several sources. HaCaT was included as an immortalized, HPV-negative, non-tumor-derived keratinocyte comparator and not as an equivalent of primary normal cervical keratinocytes.

All cell lines were cultured in RPMI-1640 (Gibco, Grand Island, NY, USA, cat. no. 11875093) medium with 10% fetal bovine serum (FBS), 1% L-glutamine, 1% penicillin, and 1% streptomycin (Gibco, cat. no. 5240-060). The culture environment was maintained at 37 °C with saturated humidity and a constant 5% CO_2_ atmosphere.

### 3.2. Protein Extraction

Cell pellets were lysed in RIPA buffer containing (10 mM Tris base (pH 7.7), 1 mM ethylenediaminetetraacetic acid (EDTA), 1% IGEPAL (a nonionic detergent), 0.1% sodium deoxycholate (SDC; Sigma-Aldrich, St. Louis, MO, USA, cat. No. 30970), and 140 mM sodium chloride (NaCl)). Each cell pellet was resuspended in 1 mL RIPA buffer, 10 µL protease inhibitor (Pierce protease inhibitor mini tablets, 1X), 10 µL Dnase I (Thermo Fisher Scientific, Waltham, MA, USA, cat. no. EN0521), 20 mM N-ethylmaleimide (a deubiquitinase inhibitor; Sigma-Aldrich, cat. no. E3876-5G), and 0.1 M dithiothreitol (DTT; Bio-Rad, Hercules, CA, USA, cat. no. 1610611). Samples were incubated overnight at room temperature to lyse the cells.

After incubation, the tubes were centrifuged at 12,000× *g* for 15 min. The supernatant was collected into a 15 mL conical tube. The pellet was resuspended in 100 µL of the previously described RIPA buffer to recover membrane proteins. The suspension was centrifuged again at 12,000× *g* for 15 min, and the supernatant was pooled with the previous extract.

The samples were then incubated at 40 °C for 30 min to reduce disulfide bonds in the proteins. Free cysteine residues were modified by adding iodoacetamide (IAA; Sigma-Aldrich, cat. no. I1149) to a final concentration of 0.2 M. The samples were then incubated at room temperature in the dark for 30 min.

To precipitate proteins, nine volumes of absolute ethanol (J.T.Baker, Phillipsburg, NJ, USA, cat. no. 9000-03) were added to the tube. The mixture was incubated at −20 °C overnight. The tubes were centrifuged at 10,000× *g* for 30 s to pellet proteins. Excess ethanol was removed. The pellet was washed three times with 90% ethanol.

### 3.3. Chemical Acetylation for Acetylation Analysis

The pellet was resuspended in deubiquitinase buffer (10 mM MES (Sigma-Aldrich, cat. no. M2933), 0.5 mM DTT (Bio-Rad, cat. no. 1610611), 50 mM Tris-HCl, and pH 7.5), and the catalytic domain of USP2 (USP2cc; Enzo Life Sciences, Farmingdale, NY, USA, cat. no. BML-UW9850) was added at a 1:50 (enzyme-to-protein) ratio to remove ubiquitin tags from lysine residues [9,18]; a related chemical strategy for ubiquitination stoichiometry has been described by Li et al. [30]. The reaction was incubated at 37 °C overnight. SDS was added to stop the reaction. Proteins were then precipitated with ethanol, as previously mentioned.

After deubiquitination, exposed amino groups were acetylated with heavy-isotope N-acetoxysuccinimide (NAS-d3) as the acetylating reagent. A 100-fold molar excess of NAS-d3 (dissolved in DMSO; Sigma-Aldrich, cat. no. D4540) relative to free amino groups was added. The reaction was incubated for 1 h at room temperature and the steps were repeated to ensure complete lysine modification. To reverse O-acetylation side reactions (on tyrosine, serine, or threonine), the samples were incubated with 5% hydroxylamine for 20 min. Excess reagents and detergents were removed by ethanol precipitation as described previously byGil, et al. [9].

### 3.4. Chemical Acetylation for Ubiquitination Analysis

Proteins were solubilized in a solution of 0.5% SDS, 0.5% SDC, and 0.1 M triethylammonium bicarbonate (TEAB Sigma-Aldrich, cat. no. T7408; pH 8.0). Free amino groups on lysine residues were acetylated with N-acetoxysuccinimide (NAS-d0). A 100-fold molar excess of NAS-d0 (dissolved in dimethyl sulfoxide (DMSO; Sigma-Aldrich, cat. no. D4540)) relative to free amino groups was added. The reaction was performed at room temperature for 1 h and repeated to ensure all free lysine residues were modified. O-acetylation side reactions were reversed as described previously. Excess reagents and detergents were removed by ethanol precipitation as described previously.

The pellet was resuspended in deubiquitinase buffer, and USP2cc (USP2cc; Enzo Life Sciences, Farmingdale, NY, USA, cat. no. BML-UW9850) (1:50) was added. This deubiquitination process was performed as described previously. After deubiquitination, newly exposed amino groups were acetylated with NAS-d3 using the same two-step protocol as for NAS-d0. Hydroxylamine treatment (5%, 20 min) was repeated to eliminate O-acetylation artifacts. Excess reagents were removed by ethanol precipitation.

NAS-d0 and NAS-d3 were synthesized in-house as described by Gil, et al. [9] and, therefore, have no commercial catalogue numbers. Their synthesis used N-hydroxysuccinimide (Sigma-Aldrich, cat. no. 130672) and acetic anhydride-d0 (Sigma-Aldrich, cat. no. 110043); or acetic anhydride-d6 (Sigma-Aldrich, cat. no. 175641), respectively. NAS-d0 and NAS-d3 were dissolved in dimethyl sulfoxide (DMSO; Sigma-Aldrich, cat. no. D4540). O-acetylation side reactions were reversed by incubation with 5% hydroxylamine (Sigma-Aldrich, cat. no. 467804) for 20 min.

### 3.5. Protein Digestion and LC-MS/MS Analysis

Modified proteins (50 µg per sample, the input used for chemical labeling) were solubilized in 0.5% SDS and 50 mM ammonium bicarbonate (ABC; Sigma-Aldrich cat. no 40867), then digested with trypsin (Promega, Madison, WI, USA, cat. no. V511) (1:50 enzyme:substrate ratio) at 37 °C for 16 h. After digestion, detergents were removed by ethyl acetate extraction [80]. The digest volume was adjusted to 100 µL with water, and one volume (100 µL) of ethyl acetate (J.T. Baker, Phillipsburg, NJ, USA, cat. no. 9003-03) was added, followed immediately by 0.5–1 µL of trifluoroacetic acid (TFA; Sigma-Aldrich, cat. no. 3022031), which turned the mixture milky. Samples were vortexed vigorously for 1–2 min and centrifuged at 13,000× *g* at room temperature, and the upper (organic) phase was discarded. The extraction was repeated once with another volume of ethyl acetate without TFA (water was added if needed to restore the aqueous volume). After centrifugation, the organic phase was removed without disturbing the interphase or the aqueous phase, and the aqueous phase was evaporated to dryness in a vacuum concentrator.

Finally, peptides were desalted using reversed-phase (RP) chromatography, dried, and stored at −80 °C until LC-MS/MS analysis.

Peptide samples were analyzed using a high-resolution LC-MS system (Dionex Ultimate 3000 RSLCnano UPLC coupled to a Q-Exactive HF-X mass spectrometer) (Thermo Fischer Scientific, Waltham, MA, USA). Data was acquired using a high-resolution variable window DIA method. Briefly, 1 µg of peptides was loaded and desalted on an Acclaim PepMap100 C18 trap column (3 µm, 100 Å, 75 µm i.d. × 2 cm) with aqueous 0.1% TFA at a flow rate of 5 µL/min for 6 min. The separation of peptides was performed on an EASY-spray RSLC C18 analytical column (2 µm, 100 Å, 75 µm i.d. × 50 cm) at a flow rate of 300 nL/min. The temperatures of the trap and analytical columns were set at 35 °C and 60 °C, respectively.

Peptides were separated using a non-linear gradient over 60 min, increasing from 2% to 27% buffer B over 55 min, then from 27% to 35% and from 35% to 55% over 2 min each, and finally reaching 90% buffer B in 1 min. The acquisition method consisted of a full acquisition cycle comprising 54 variable-width MS2 scans and 3 MS1 scans over the mass range of 385–1460 *m*/*z*. The MS2 window width was determined based on the empirical distribution of signals from DDA runs with similar samples, with an overlap of 1 Da between adjacent windows.

The parameters for MS1 were as follows: resolution of 120,000 (@200 *m*/*z*), AGC target of 3 × 10^6^, and maximum injection time of 50 ms. For MS2, the resolution was set to 30,000, with an AGC target of 1 × 10^6^, an automatic maximum injection time, and an NCE of 28.

### 3.6. Raw Data Analysis with DIA-NN

DIA-NN version 2.0 [81] was used to search DIA raw files for precursor and fragment identifications based on two-dimensional peak positions (retention time and *m*/*z* precursor) (DIA-NN (version 2.0) is freely available academic software (Demichev laboratory, Berlin, Germany; https://github.com/vdemichev/DiaNN) (accessed on 7 May 2025).) following the multiplexed-DIA strategy of Thielert et al. [82], using AlphaPeptDeep-predicted spectral libraries (acetyl labeled tryptic H sapiens) (AlphaPeptDeep (version 1.2.1) is open-source software developed by Mann Labs, at the Max Planck Institute of Biochemistry, Martinsried, Germany (https://github.com/MannLabs/alphapeptdeep) (accessed on 27 April 2025)).

The DIA-NN search parameters included Proteotypicity = “Genes”, Quantification strategy = “QuantumUMS (high-precision)”, Cross-run normalization = “RT-dependent”, Library Generation = “IDs, RT and IM Profiling”, and Speed and RAM usage = “Optimal results”. Additionally, “MBR” and “Protein inference” settings were enabled. Trypsin was specified as the protease, and cysteine carbamidomethylation was set as a fixed modification. The maximum number of variable modifications was set to 2, with methionine oxidation and N-terminal acetylation considered as variable modifications. The peptide length range was set to 7–30 amino acids, precursor charge range to 1–4, precursor *m*/*z* to 300–1800, and fragment ion *m*/*z* range to 200–1800. For acetyl labeled samples, the following additional commands were entered into the DIA-NN command line GUI: (1) {--fixed-mod Acetyl, 42.010565, nK}, (2) {--channels Acetyl,0,K,0:0; Acetyl,3,K,3.018:3.018}, (3) {--original-mods}, (4) {--channel-spec-norm}, (5) {--peak-translation}, (6) {--site-ms1-quant}, and (7) {--report-lib-info}. The UniProt is a database of reviewed *Homo sapiens* sequence was used (UP000005640, https://www.uniprot.org/proteomes/UP000005640, (accessed on 1 May 2025)).

### 3.7. AlphaPeptDeep Library Prediction

Libraries were predicted by AlphaPeptDeep [83] using the same FASTA file, as described above. Predefined standard models of MS2, RT and CCS were used without transfer learning; MaxQuant was not used, and CCS values were predicted only, since the Q Exactive HF-X does not measure ion mobility. AlphaPeptDeep generated the predicted library, whereas DIA-NN analyzed the experimental DIA files; AlphaPeptDeep was chosen because it allows lysine acetylation to be set as a fixed label. Peptide labeling was set to “Acetyl@K” and “Acetyl@Any N-term” with a mass of 42.0106 (∆0). The library only needs to be predicted for one channel (in our case ∆0, as used for the reference channel), since DIA-NN creates the library for the other channels automatically using the additional commands. All other settings were used as standard. In short, “None” was used as the decoy method and trypsin was selected as the protease. A maximum of one missed cleavage of 1 was allowed. “Carbamidomethyl@C” was set as a fixed modification and “Oxidation@M” as a variable modification, with a maximum of one variable modification. The peptide length was set from 7 to 30, the precursor charge from 1 to 4 and the precursor *m*/*z* from 200 to 1800. b- and y-ions were used as fragment types with a maximum fragment charge of 2. The top 12 fragments per precursor were kept from 200 to 2000 *m*/*z*. Predicted RT was converted to iRT and a TSV file was exported for the DIA-NN search.

### 3.8. Data Analysis

The DIA-NN parquet report was analyzed using R version 4.5 (R Foundation for Statistical Computing, Vienna, Austria; https://www.r-project.org) and Python 3.14 (pandas, SciPy and statsmodels). To calculate MaxLFQ abundance for protein groups, we used the DIA-NN R package. For MS2-centric methods, MaxLFQ abundance was based on the “Precursor.Normalised” column, whereas for MS1-centric methods, it was based on the “Ms1.Area” column. For each precursor, the reference channel (d3 in the acetylation workflow and d0 in the ubiquitination workflow) was filtered for Channel.Q.Value < 0.01, PG.Q.Value < 0.05 and Global.PG.Q.Value < 0.01; the low-abundance channel was quantified by DIA-NN peak translation and required a non-zero MS1 area.

After obtaining filtered data from DIA-NN, we selected Channel.0 and Channel.3, corresponding to the light (d_0_) and heavy (d_3_) channels, respectively, representing MS1 isotope intensities, from the Channel column. In the acetylation workflow, d_0_ corresponds to endogenous acetylation; in the ubiquitination workflow, d_0_ contains endogenous acetylation and chemically blocked lysines, and d_3_ corresponds to lysines exposed by USP2cc. Only precursors with non-zero values in both channels were retained, charge states of the same peptide were summed, and the lowest 10% of total MS1 area per run was removed. We also selected peptides present in all three replicates of each cell line. Using the formula established by Baeza et al. [17], acetylation stoichiometry for each peptide was calculated:

Stoichiometry (%) = [endogenous/(endogenous + heavy)] × 100

Ubiquitination stoichiometry for each peptide was calculated using the following formula:

Stoichiometry (%) = [heavy/(endogenous + heavy)] × 100

Three biological replicates (independent cultures, one injection each, and no technical replicates) were performed for each cell line. Stoichiometry data are presented as mean ± standard deviation. To identify statistically significant differences in stoichiometry across experimental groups, we performed a one-way analysis of variance (ANOVA) in R (version 4.5; R Foundation for Statistical Computing, Vienna, Austria), with multiple testing correction applied using the Benjamini–Hochberg method to control the false-discovery rate (FDR). For each modified peptide quantified in all three replicates of at least two cell lines, one-way ANOVA compared peptide-level stoichiometry values across those cell lines, independently for the acetylome and the ubiquitinome datasets, and Tukey HSD post hoc tests were applied to every significant peptide. Peptides with an adjusted *p*-value < 0.05 were considered statistically significant. To visualize abundance patterns among differentially regulated peptides, protein-level stoichiometry (mean of the modified peptides of each protein per replicate) was used, keeping proteins with fewer than 50% missing values across the 12 runs. For the heatmap, the 150 proteins with the highest variance were selected, remaining missing values were imputed with the protein median, and values were z-scored by row. The number of clusters was selected with the gap statistic (clusGap with k-means, nstart = 25, B = 50, K.max = 10, maxSE rule, and seed 123; k limited to 2–6); rows were split by k-means clusters, ordered within each cluster by hierarchical clustering (Euclidean distance and complete linkage), and columns were ordered by cell line. PCA was performed on all eligible proteins (prcomp, scale = TRUE); a PCA restricted to proteins quantified in all runs, without imputation, is shown in Figure S2. Shared lysines were defined as single-lysine peptides quantified in all three replicates of at least one cell line in both workflows. Spearman correlations between enzyme abundance (MaxLFQ) and run-level median stoichiometry were computed across the 12 biological samples for enzymes quantified in at least six runs, with Benjamini–Hochberg correction.

### 3.9. Functional-Enrichment Analysis

Functional-enrichment analysis of uniquely modified proteins identified per cell line was performed in Metascape (https://metascape.org (accessed on 19 June 2025)), querying Gene Ontology (GO), KEGG (https://www.genome.jp/kegg/ (accessed on 19 June 2025)) and Reactome (https://reactome.org accessed on 21 June 2025) pathway annotations; terms with an adjusted *p*-value < 0.05 were considered significantly enriched. Protein–protein interaction networks were retrieved from the STRING (https://string-db.org) database and visualized and analyzed in Cytoscape (version 3.10.3; Cytoscape Consortium; https://cytoscape.org).

### 3.10. Availability of Data and Materials

The mass-spectrometry proteomics data generated in this study have been deposited in the ProteomeXchange Consortium through the PRIDE partner repository under the dataset identifier PXD076171 and are available at https://www.ebi.ac.uk/pride/archive/projects/PXD076171. The deposited files include the raw mass-spectrometry data, sample metadata, sequence database and spectral-library inputs, DIA-NN parameters and output reports, and the processed protein-, peptide-, modification-site-, and stoichiometry-level quantitative data.

### 3.11. Declaration of Generative AI Use

During the preparation of this work, the authors used AI tools (Claude Sonnet 4.5 (Anthropic, San Francisco, CA, USA)) to improve the readability and language of the manuscript or to generate images. The authors subsequently reviewed and edited the AI-generated content as necessary and take full responsibility for the final content of this manuscript.

## 4. Conclusions

**Figure 6:**
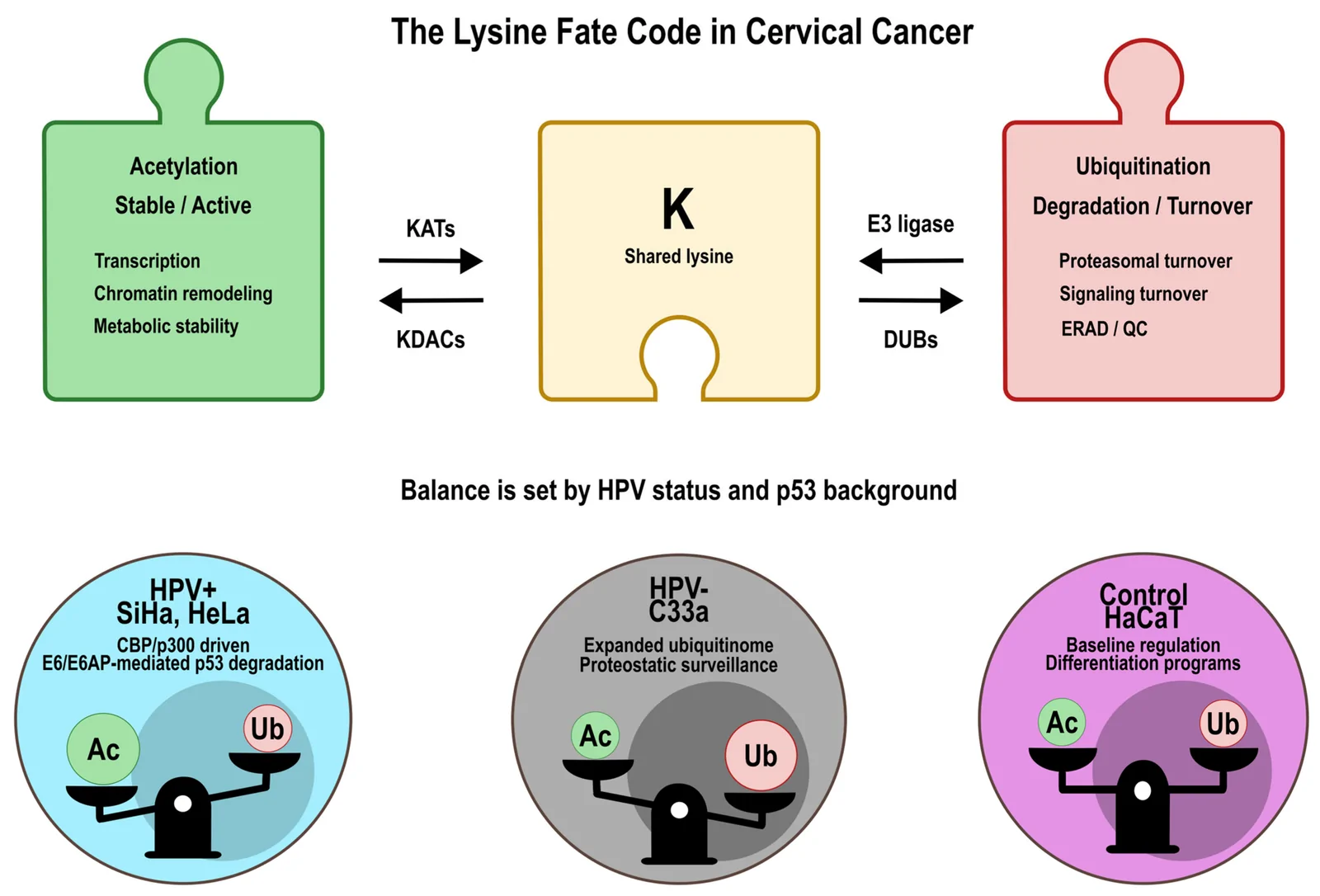
Schematic model of the lysine-fate code in cervical cancer (hypothetical). A shared lysine residue is subject to competing modification by lysine acetyltransferases/deacetylases (KATs/KDACs) and E3 ubiquitin ligases/deubiquitinases (DUBs). High-occupancy acetylation proposed to favor a stable, functionally active protein state (transcriptional activity, chromatin remodeling, and metabolic stability), whereas ubiquitination favors degradation or turnover (p53 clearance, innate immune suppression, and ERAD/quality control). The balance between these outcomes is proposed to depend on cellular context: HPV-positive lines (SiHa and HeLa) share differences in acetylation, possibly linked to CBP/p300 signaling; the HPV-negative C33A line shows a larger ubiquitinome; and the non-tumorigenic HaCaT control shows a more balanced, baseline regulatory architecture reflecting differentiation programs. Created with Inkscape 1.4 (https://inkscape.org).

Collectively, this study shows that protein acetylation and ubiquitination are interconnected regulatory systems. Their patterns differ among cervical cell lines with different HPV statuses and p53 mutational background. Using a DIA-MS-based stoichiometric quantification approach, we identified distinct cell line-specific PTM architectures. HPV-positive cells shared differences in acetylation of transcriptional and chromatin-associated proteins. In contrast, the HPV-negative C33A line showed a larger number of quantified ubiquitinated peptides, which may relate to proteostasis and ERAD. These differences suggest that lysine residues may act as competitive regulatory nodes that influence whether proteins remain functionally active or are directed toward degradation. The identification of 168 acetylated and 76 ubiquitinated peptides with significant cell line-dependent PTM differences advances the understanding of cervical cancer heterogeneity and provides a valuable resource for biomarker discovery. Moreover, differential modification of proteins such as TOM1, WDR43, SQOR, IFIT3, DDX3X and CTPS1 points to candidate links with metabolism, antiviral immunity, vesicular trafficking, and epigenetic programs that remain to be tested. These findings are consistent with the proposed “lysine-fate code,” in which acetylation and ubiquitination occupancy may contribute to proteome-wide decisions (Figure 6).

Taken together, this work advances the field in three ways. Methodologically, it introduces a multiplexed DIA-MS chemical-labeling workflow that quantifies acetylation and ubiquitination stoichiometry side by side [17,22,30,75,81]. Conceptually, it proposes that the relative occupancy of acetylation versus ubiquitination at shared lysine residues, rather than the mere presence of either mark, contributes to protein fate. This proposed “lysine-fate code” [19,66,67], requires testing through perturbation experiments. Biologically, it provides an integrated acetylome/ubiquitinome stoichiometric dataset across HPV-positive, HPV-negative and non-tumorigenic cervical models, describing PTM differences among models that differ in terms of HPV status and p53 mutational background and nominating testable therapeutic strategies: proteasome inhibition for the ubiquitination-dominant C33A state, and HDAC inhibition for the acetylation-dominant HeLa state.

Overall, this work provides new insights into cervical cancer’s molecular basis. It also suggests PTM-regulating enzymes as therapeutic vulnerabilities and proposes a conceptual framework for precision oncology strategies aimed at reprogramming the balance between protein stability and degradation.

## Figures and Tables

**Table 1:** Summary of acetylated and ubiquitinated peptide and protein identifications across cell lines.

Cell Line	HPV Status	Acetylation				Ubiquitination			
		Total Peptides (Mean ± SD)	Total Proteins (Mean ± SD)	Modified Peptides	Modified Proteins	Total Peptides (Mean ± SD)	Total Proteins (Mean ± SD)	Modified Peptides	Modified Proteins
SiHa	HPV16+	37,638 ± 931	6648 ± 122	522	462	32,278 ± 650	6167 ± 74	2326	1401
HeLa	HPV18+	39,181 ± 1088	6666 ± 111	814	704	33,678 ± 802	6177 ± 73	2694	1442
C33A	HPV−	40,509 ± 1744	6799 ± 153	673	594	35,048 ± 655	6383 ± 70	4987	2043
HaCaT	Control	38,085 ± 479	6668 ± 60	561	495	31,860 ± 782	6091 ± 94	2902	1475

## Data Availability

The mass spectrometry proteomics data are available via the ProteomeXchange Consortium through the PRIDE partner repository, dataset identifier PXD076171 (https://www.ebi.ac.uk/pride/archive/projects/PXD076171). Any additional requests can be directed to the corresponding author.

## Supplementary Materials

The following supporting information can be downloaded at https://www.mdpi.com/article/10.3390/ijms27219749/s1.

## Abbreviations

The following abbreviations are used in this manuscript:

PTM	post-translational modification
DIA-MS	data-independent acquisition mass spectrometry
HPV	human papillomavirus
KAT	lysine acetyltransferase
KDAC	lysine deacetylase
DUB	Deubiquitinase
UPS	ubiquitin-proteasome system
ANOVA	analysis of variance
FDR	false-discovery rate
PCA	Principal-component analysis
ERAD	endoplasmic reticulum-associated degradation
NAS	N-acetoxysuccinimide
USP2cc	catalytic domain of ubiquitin-specific protease 2
