# Supplementary material for: Comparative Acetylation and Ubiquitination Profiling in Cervical Cancer-Cell Models Supports a Proposed Lysine-Fate Code
Source: Int J Mol Sci. 2026 Oct 30;27(21):9749. doi: 10.3390/ijms27219749 (PMC13762473; doi:10.3390/ijms27219749)
Supplement: Supplementary file 1 [file ijms-27-09749-s001.zip › ijms-4620231-supplementary.pdf]

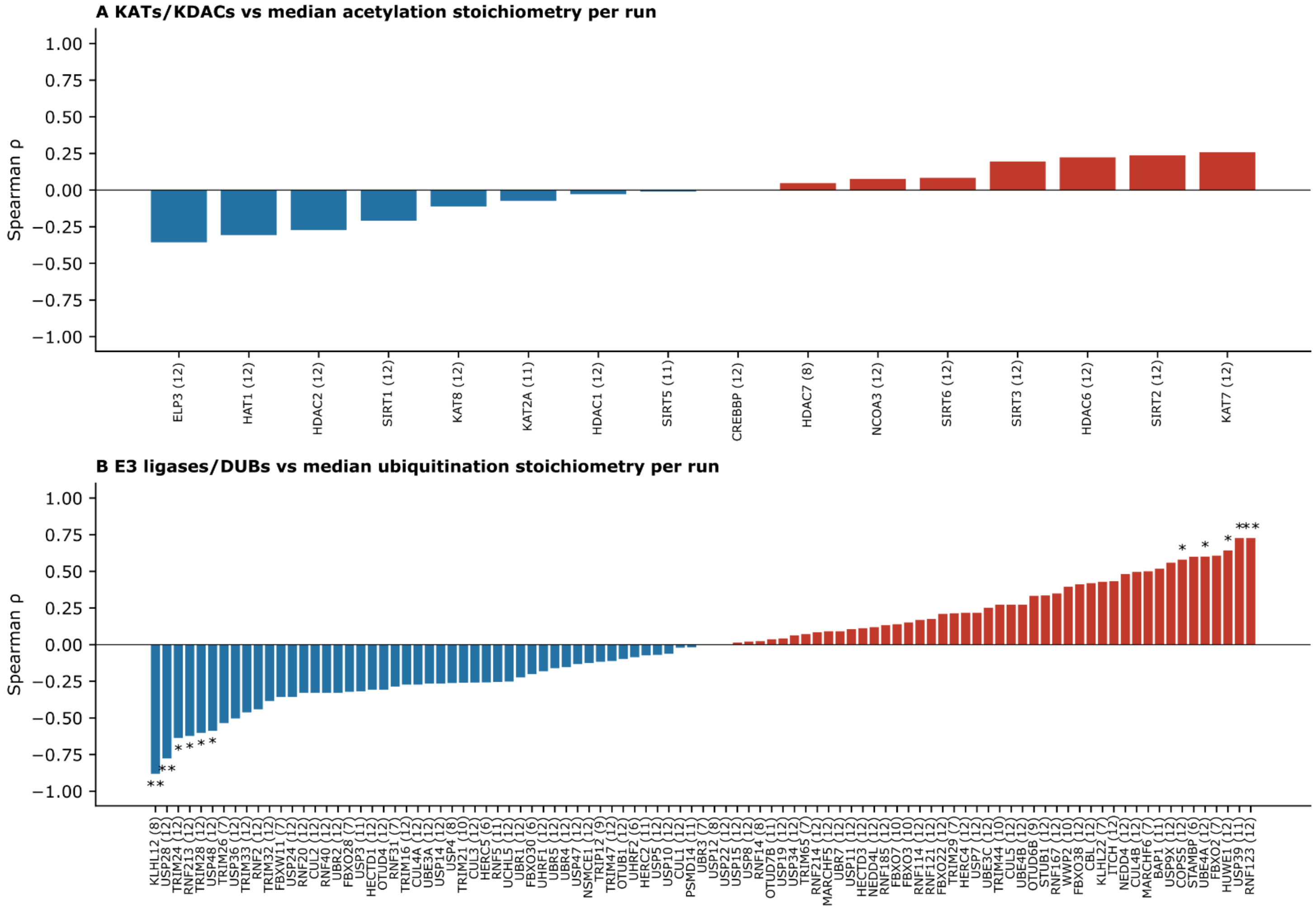

Figure S1: Correlation between enzyme abundance and PTM stoichiometry. A) Lysine acetyltransferases and deacetylases (KATs/KDACs, n = 16) versus the median acetylation stoichiometry of each run. B) E3 ubiquitin ligases and deubiquitinases (E3s/DUBs, n = 97) versus the median ubiquitination stoichiometry of each run. Bars show the Spearman correlation coefficient (ϱ) between log2 MaxLFQ protein abundance and run-level median stoichiometry across the 12 biological samples. Red bars indicate positive correlations and blue bars negative ones. Only enzymes quantified in at least six runs were included, and the number of runs is shown in parentheses after each name. Asterisks mark nominal p-values (* $p < 0.05$, ** $p < 0.01$, *** $p < 0.001$)

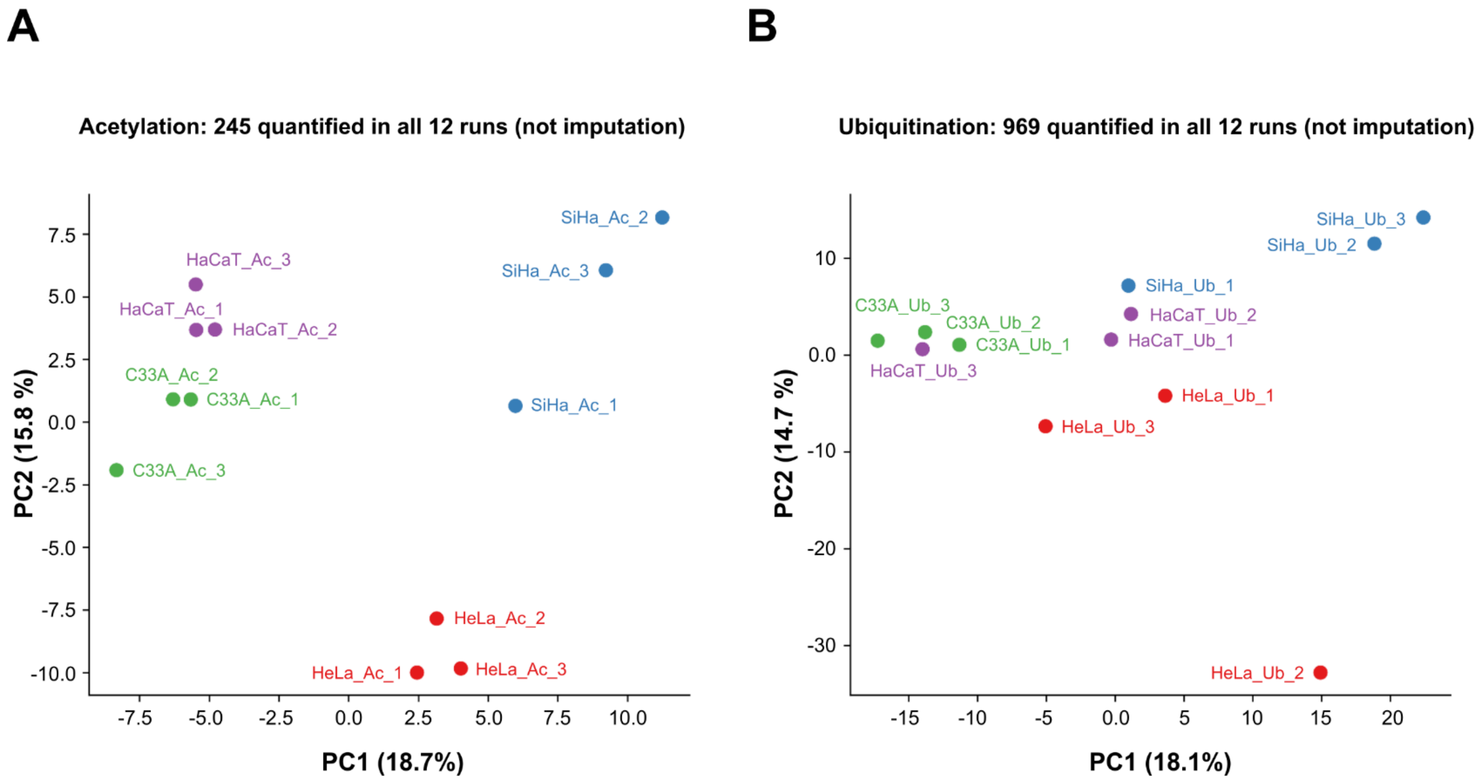

Figure S2. Principal component analysis without imputation. A) Acetylation: 245 proteins quantified in all 12 runs (PC1 18.7%, PC2 15.8%). B) Ubiquitination: 969 proteins quantified in all 12 runs (PC1 18.1%, PC2 14.7%). The input was protein-level occupancy, the mean of each protein's modified peptides. Values were scaled to unit variance before PCA. Points are individual biological replicates colored by cell line.

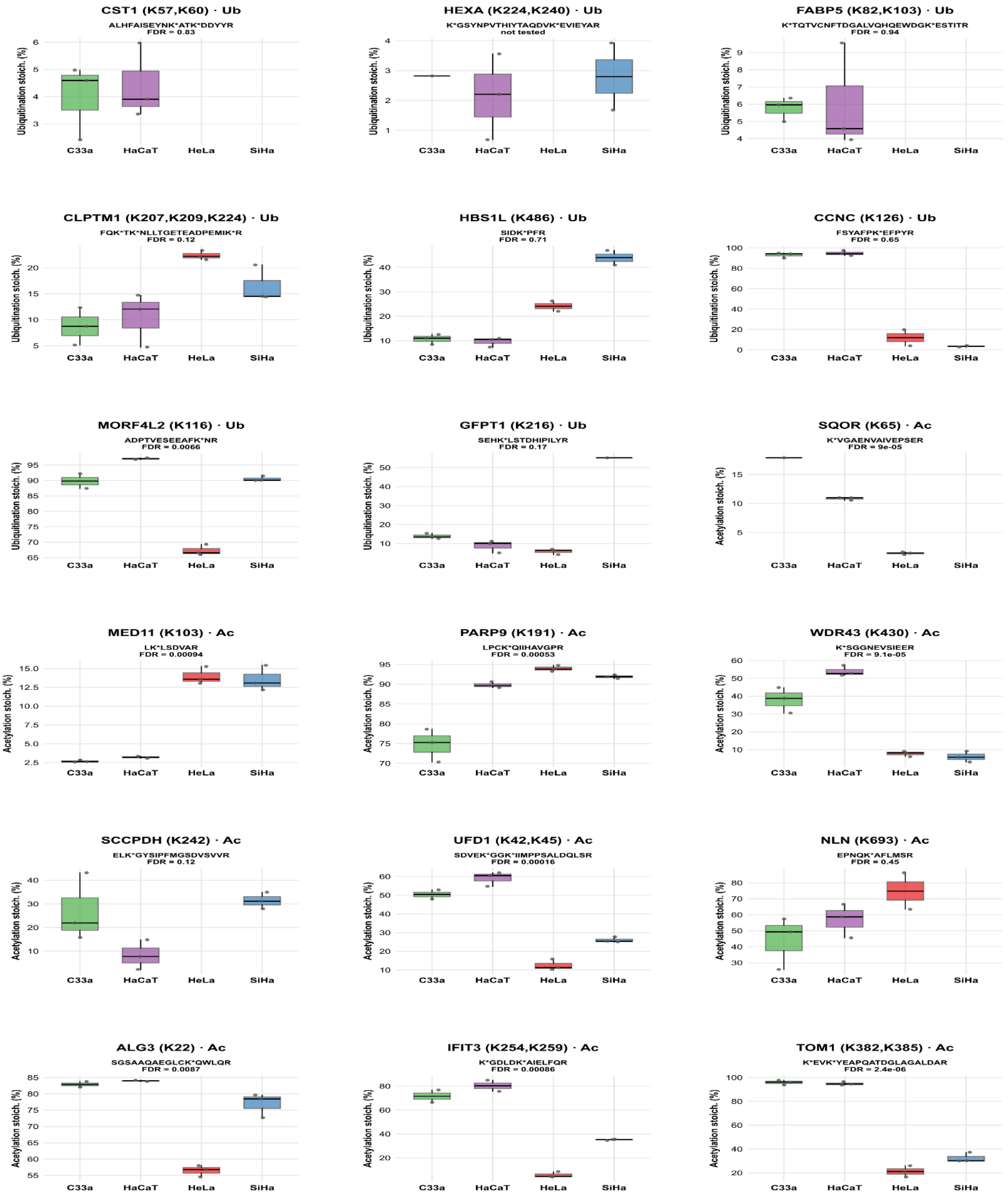
CST1 (K57,K60) · Ub
ALHFAISEYNK*ATK*DDYYR
FDR = 0.83
HEXA (K224,K240) · Ub
K*GSYNPVTHIYTAQDVK*EVIEYAR
not tested
FABP5 (K82,K103) · Ub
K*TQTVCNFTDGALVQHQEWDGK*ESTITR
FDR = 0.94
CLPTM1 (K207,K209,K224) · Ub
FQK*TK*NLLTGETEADPEMIK*R
FDR = 0.12
HBS1L (K486) · Ub
SIDK*PFR
FDR = 0.71
CCNC (K126) · Ub
FSYAFPK*EFPYR
FDR = 0.65
MORF4L2 (K116) · Ub
ADPTVESEEAFK*NR
FDR = 0.0066
GFPT1 (K216) · Ub
SEHK*LSTDHIPILYR
FDR = 0.17
SQOR (K65) · Ac
K*VGAENVAIVEPSER
FDR = 9e-05
MED11 (K103) · Ac
LK*LSDVAR
FDR = 0.00094
PARP9 (K191) · Ac
LPCK*QIIHAVGPR
FDR = 0.00053
WDR43 (K430) · Ac
K*SGGNEVSIEER
FDR = 9.1e-05
SCCPDH (K242) · Ac
ELK*GYSIPFMGSDVSVVR
FDR = 0.12
UFD1 (K42,K45) · Ac
SDVEK*GGK*IIMPPSALDQLSR
FDR = 0.00016
NLN (K693) · Ac
EPNQK*AFLMSR
FDR = 0.45
ALG3 (K22) · Ac
SGSAAQAEGLCK*QWLQR
FDR = 0.0087
IFIT3 (K254,K259) · Ac
K*GDLDK*AIELFQR
FDR = 0.00086
TOM1 (K382,K385) · Ac
K*EVK*YEAPQATDGLAGALDAR
FDR = 2.4e-06
Ubiquitination stoich. (%)
Acetylation stoich. (%)
C33a
HaCaT
HeLa
SiHa

Figure S3. Replicate-level stoichiometry of candidate proteins. Ubiquitination: CST1, HEXA, FABP5, CLPTM1, HBS1L, CCNC, MORF4L2 and GFPT1. Acetylation: SQOR, MED11, PARP9, WDR43, SCCPDH, UFD1, NLN, ALG3, IFIT3 and TOM1. For each protein, the peptide quantified in the most replicates is shown. The panel title gives the gene, lysine position, peptide sequence and FDR of the one-way ANOVA (Supplementary Tables S1–S2).
